# Identification of biological signatures of cruciferous vegetable consumption utilizing machine learning-based global untargeted stable isotope traced metabolomics

**DOI:** 10.3389/fnut.2024.1390223

**Published:** 2024-07-03

**Authors:** John A. Bouranis, Yijie Ren, Laura M. Beaver, Jaewoo Choi, Carmen P. Wong, Lily He, Maret G. Traber, Jennifer Kelly, Sarah L. Booth, Jan F. Stevens, Xiaoli Z. Fern, Emily Ho

**Affiliations:** ^1^School of Nutrition and Public Health, Oregon State University, Corvallis, OR, United States; ^2^Linus Pauling Institute, Oregon State University, Corvallis, OR, United States; ^3^Department of Electrical Engineering and Computer Science, Oregon State University, Corvallis, OR, United States; ^4^Jean Mayer USDA Human Nutrition Research Center on Aging, Tufts University, Boston, MA, United States; ^5^Department of Pharmaceutical Sciences, Oregon State University, Corvallis, OR, United States

**Keywords:** cruciferous vegetables, metabolomics, stable isotope tracing, machine learning, precision nutrition

## Abstract

In recent years there has been increased interest in identifying biological signatures of food consumption for use as biomarkers. Traditional metabolomics-based biomarker discovery approaches rely on multivariate statistics which cannot differentiate between host- and food-derived compounds, thus novel approaches to biomarker discovery are required to advance the field. To this aim, we have developed a new method that combines global untargeted stable isotope traced metabolomics and a machine learning approach to identify biological signatures of cruciferous vegetable consumption. Participants consumed a single serving of broccoli (*n* = 16), alfalfa sprouts (*n* = 16) or collard greens (*n* = 26) which contained either control unlabeled metabolites, or that were grown in the presence of deuterium-labeled water to intrinsically label metabolites. Mass spectrometry analysis indicated 133 metabolites in broccoli sprouts and 139 metabolites in the alfalfa sprouts were labeled with deuterium isotopes. Urine and plasma were collected and analyzed using untargeted metabolomics on an AB SCIEX TripleTOF 5,600 mass spectrometer. Global untargeted stable isotope tracing was completed using openly available software and a novel random forest machine learning based classifier. Among participants who consumed labeled broccoli sprouts or collard greens, 13 deuterium-incorporated metabolomic features were detected in urine representing 8 urine metabolites. Plasma was analyzed among collard green consumers and 11 labeled features were detected representing 5 plasma metabolites. These deuterium-labeled metabolites represent potential biological signatures of cruciferous vegetables consumption. Isoleucine, indole-3-acetic acid-N-O-glucuronide, dihydrosinapic acid were annotated as labeled compounds but other labeled metabolites could not be annotated. This work presents a novel framework for identifying biological signatures of food consumption for biomarker discovery. Additionally, this work presents novel applications of metabolomics and machine learning in the life sciences.

## 1 Introduction

Interest in biomarker discovery has grown tremendously over the last decade with biomarkers of disease and exposures being developed across the life sciences (1–5). Discovery of biological signatures of specific foods and dietary patterns is of interest because of the potential of food biomarkers to improve our understanding of the role of nutrition in the etiology and prevention of disease (6–9). Liquid chromatography-mass spectrometry based metabolomics presents a powerful approach to conduct metabolite-based biological signature and biomarker discovery (10–12). Untargeted metabolomics allows investigators to utilize a data-driven approach to identify compounds that are associated with the biological question under investigation, without relying on *a priori* knowledge to guide data analysis. Traditional untargeted metabolomics approaches to biomarker discovery identify metabolomic features associated with a specific food or other environmental exposures. However, these techniques are unable to differentiate between metabolites produced by the exposed individual (host-derived metabolites) and metabolites that originate directly from the exposure (10, 13). As a result, discovered signatures and biomarkers from these methods may not be specific to the exposure of interest and instead may be endogenous compounds that respond to an exposure (effect biomarkers) (7). As effect biomarkers are produced by the host they could also be influenced by physiological status and other confounding dietary components, making effect biomarkers potentially non-specific to the exposure of interest. Likewise, other foods that the participant consume during the study period may also alter the participant’s metabolome (both through direct metabolites from the food or by changing the host’s endogenous metabolome) adding difficulty in identifying clear signatures of consumption. These problems highlight a need for more advanced methodologies that directly associate potential biomarkers with the specific food that is under investigation (6). An ideal food biomarker is not present prior to ingestion of the food and has a measurable time- and dose-dependent response to consumption (7, 12).

Global untargeted stable isotope traced metabolomics uses substrates like foods labeled with relatively-rare heavy isotopes, such as ^13^C and ^2^H, offering a possible innovative tool to address the problem of non-specific biomarker discovery. Stable isotope tracing is achieved in the nutrition field by introducing the isotope (like ^2^H) during production of the food of interest and creating a labeled food. Labeled vegetables can be generated by growing them with deuterium-oxide (D_2_O) mixed into the water. This results in the molecules in the labeled vegetable having a distinct and detectable mass isotopologue distribution. An isotopologue is defined as a molecule that has an identical structure and chemical formula, but differs only in isotopic composition. In labeled vegetables, the resulting mass isotopologue distribution is unique to the labeled food and importantly differs from the isotopologue distribution of endogenous and non-labeled metabolites. After consumption of the labeled food, labeled molecules can be detected in participants’ urine and plasma samples via global untargeted stable isotope traced metabolomics. The presence of the label in the metabolite of interest facilitates the determination if the metabolite is from the food of interest (labeled metabolite) or present in the participant independent of vegetable consumption (unlabeled metabolite). The resulting identified metabolic signatures of consumption are potential biomarker candidates for further validation and quantification. Stable isotope tracing has been in use for years but its application has been limited to looking for specific metabolites of a labeled compound in a known metabolic pathway (13–15). Recent advances now allow for global untargeted stable isotope tracing which is an important advance in biomarker discovery because compounds derived from foods and microbes are often unannotated (16–18). Currently many tools for global untargeted stable isotope traced metabolomics, including software like X^13^CMS, geoRge, and HiResTEC, have been developed and tested specifically on cell culture models that are relatively simple and well controlled systems (19–23). These software platforms compare isotopologue distributions from labeled samples against unlabeled controls to identify stable-isotope labeled metabolites. In human biological samples, endogenous metabolism of labeled-compounds can dilute the abundance of labeled compounds and decrease their degree of labeling. This issue coupled with the complexity of untargeted metabolomics (ion suppression, and a high number of coeluting peaks) leads to a high number of false positive and false negative results from these currently available software tools. Thus, there is a pressing need for the development of new tools for use in complex biological systems and machine learning modeling can address this need by quickly ranking candidates for manual curation and validation (24).

To address this need, our objective was to conduct global untargeted stable isotope tracing in human biological samples and develop a machine learning approach to classify candidate labeled-metabolites detected by HiResTEC as labeled or unlabeled. We conducted this work using biological samples from human subjects fed labeled cruciferous vegetables (collard greens or broccoli sprouts) or control (alfalfa sprouts). Cruciferous vegetables are widely studied because they contain compounds that are known to have chemopreventive and cancer-suppressive properties (25, 26). Increased consumption of cruciferous vegetables has been inversely associated with the risk of developing prostate, breast, colorectal, lung, bladder, gastric, pancreatic and renal cancer and cruciferous vegetables may be beneficial in preventing other chronic diseases (27, 28). The development of methods to identify biological signatures of cruciferous vegetables consumption could aid in further understanding the health benefits of cruciferous vegetables, and in advancing the field of precision nutrition. Our approach performed successfully on metabolomics data from human urine and human plasma, generated from two different feeding studies, utilizing cruciferous vegetables grown for different periods of time with D_2_O. This work presents a novel approach to discovering biological signatures of food consumption and demonstrates the feasibility of utilizing global untargeted stable isotope tracing to identify stable-isotope enriched metabolites derived from foods in humans.

## 2 Materials and methods

### 2.1 Study summary

Biological samples (urine and plasma) were collected from two different human feeding studies that were previously published: (1) broccoli sprouts feeding study (includes a non-cruciferous vegetable control arm where participant consumed alfalfa sprouts) (NCT04641026) (29), and (2) collard greens feeding study with collard greens as a source of phylloquinone (vitamin K) (NCT00336232) (30). We also utilized the vegetable material from these studies to train our machine learning approach. This included labeled and unlabeled 6-day old broccoli sprouts, 6-day old alfalfa sprouts, and 3 month old collard greens. All human study protocols were approved by the Oregon State University Institutional Review Board (IRB-2019-0123, IRB8343) and the Tufts University Institutional Review Board (IRB7421). All subjects provided written informed consent prior to being enrolled into each study.

### 2.2 Cultivation of labeled vegetables

Broccoli sprouts, alfalfa sprouts and collard greens were grown in the presence (labeled) or absence (unlabeled) of D_2_O. Broccoli and alfalfa sprouts were grown from commercially available seed (Sprout House, Kingston, NY and True Leaf Market, Salt Lake City, UT). Sprout seeds were sanitized with calcium hypochlorite (20,000 ppm, 15 min), rinsed, and then soaked overnight in H_2_0 or 25% D_2_O as previously described (31–37). Sprouts were then rinsed twice daily with H_2_0 or 25% D_2_O for 5 additional days, harvested on day 6 and refrigerated until use. For labeled collard greens, we took advantage of archived samples from a separate trial conducted at Tufts University. Collard greens were grown hydroponically in the presence of 31% D_2_O as previously described (30) and growing conditions varied because the vegetables are consumed at differing ages of maturity. Unlabeled collard greens were purchased locally from First Alternative Co-Op (Corvallis, OR) and sourced from Winter Green Farm (Noti, OR).

### 2.3 Human study designs

#### 2.3.1 Broccoli spout study

For the broccoli study, thirty two healthy women and men, 19–55 years old, were recruited in Corvallis, Oregon (29). The study was conducted in the Linus Pauling Institute and the Moore Family Center metabolic kitchen between April and November 2021. Exclusion criteria included (1) tobacco use; (2) BMI <18.5 or > 30.0 kg/m2; (3) pregnancy or breastfeeding; (4) use of oral antibiotic medication (within past 6 months); (5) extensive vigorous exercise (7+ hours per week); (6) use of medications to control cholesterol levels or fat absorption; and (7) a history of significant acute or chronic illness and, bariatric surgery and, gastrointestinal procedures or disorders. Eligibility of subjects was confirmed. Subjects were randomized to four treatment groups, receiving either (1) unlabeled broccoli sprouts, (2) labeled broccoli sprouts, (3) unlabeled alfalfa sprouts, or (4) labeled alfalfa sprouts. The study was organized as 8 cohorts and there were no differences between treatment groups in age (mean age 33), sex (59% female, 41% male), nor racial composition (50% White, 28% Asian, and the remaining 22% included person identifying as American Indian/Alaskan Native, African American, more than one race, other race, or decline to answer).

Participants in the broccoli arms consumed fresh broccoli sprouts (40.5 g on average) containing 100 μmol sulforaphane equivalents. Sulforaphane contents in broccoli sprouts were analyzed on the day of harvest for every cohort as previously described (29). The alfalfa sprout dose was equivalent in weight to the amount of sprouts consumed by the broccoli sprout participants. Participants in all arms consumed sprouts with a standardized breakfast and fasted for at least 8 h prior to the meal (32). One week before and throughout the sample collection period, subjects self-reported dietary intake and were instructed to avoid consuming foods, beverages, and supplements containing cruciferous vegetables, and live/active cultures, or probiotics. Participant intake records indicated compliance with avoiding confounding food items. Diet records were analyzed using Food Processor^®^ SQL (EHSA, Salem, OR).

Baseline 0 h spot urine collections were obtained prior to sprout consumption. Following consumption of sprouts, total urine was collected over 72 h with urine collections occurring between 0–3, 3–6, 6–24, 24–48, and 48–72 h post consumption. While in the subject’s possession, urine was refrigerated or kept on ice in opaque jugs containing granulated boric acid (~20 mg/mL) to stabilize metabolites. Upon receipt, the urine was acidified with trifluoroacetic acid (TFA) to a final concentration of 10% v/v, frozen in liquid nitrogen, and stored at -80C until analysis.

#### 2.3.2 Collard greens study

The collard greens study took advantage of unique archived urine and plasma samples that were collected pre- and post- consumption of heavily labeled collard greens (30). These samples were generated when 21 participants, 18–40 year old, resided at the Metabolic Research Unit (MRU) at JM USDA HNRC at Tufts University and consumed a diet low in cruciferous vegetables. The diet was provided on a rotating menu every 3 days for 1 month. On day 28 participants consumed a single dose of 100 g steamed deuterium-labeled collard greens with breakfast. Total 24 h urine was collected during the cruciferous vegetable depletion phase (control urine) or during the 24 h following collard green consumption (labeled urine). Control plasma was collected at 0 h and labeled plasma was collected 4 h post-labeled collard greens consumption. Details of how urine and plasma samples were processed are previous published (30). Because HiResTEC software requires unlabeled control samples to do global untargeted stable isotope tracing, we conducted a small trial (*n* = 5, Corvallis, Oregon) to generate the needed unlabeled samples. Like the urine and plasma samples from the archived collard green samples, healthy adult participants consumed 100 g of steamed collard greens, although the collard greens were unlabeled. The methods of plasma and urine collection replicated those at Tufts University (30).

### 2.4 Metabolomics analysis

**Figure 1:**
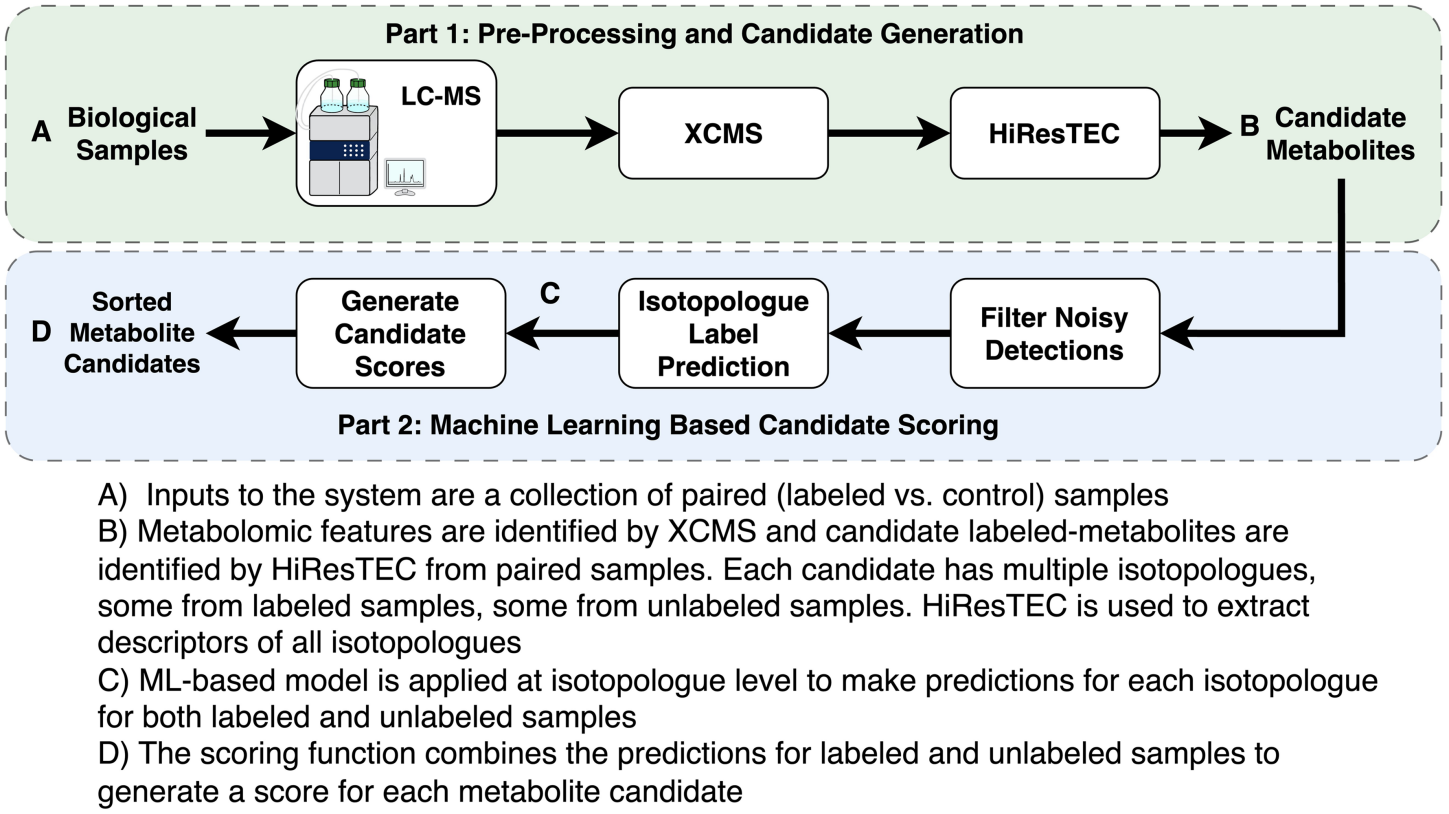
Description of analysis pipeline.

The same metabolomics method was used on all samples, however, extraction protocols differed (see below). Briefly, HPLC was performed on a Shimadzu Nexera system with a phenyl-3 stationary phase column (Inertsil Phenyl-3, 5 μm, 4.6 × 150 mm, GL Sciences) coupled to a quadrupole time-of-flight MS (AB SCIEX TripleTOF 5,600), as previously described (38, 39) (Figure 1A).

Metabolomics data preprocessing is described in Figure 1. Preprocessing of metabolomic features was completed using XCMS (v3.12.0) (40–42) Optimal preprocessing parameters for XCMS were selected using AutoTuner (v1.4.0) (43). XCMS preprocessing parameters are shown in Supplementary Table S1. HiResTEC (v0.59) was used to identify candidates to evaluate with our machine learning (ML) model (23) (Figure 1B). HiResTEC was chosen over X^13^CMS and geoRge as it has been shown to perform the best of the three packages (44). Metabolite annotation was conducted using Canopus for *de novo* annotation, or using our in-house metabolite library, or manual interpretation of MS/MS fragmentation patterns (45).

#### 2.4.1 Human biofluid metabolite extraction

Metabolites from urine were extracted (100 μL urine/400 μL ice cold methanol), mixed vigorously, and clarified by centrifugation (14,000 rpm for 5 min) then transferred to MS vials (46). Metabolites from plasma were extracted (50 μL plasma/200 μL ice cold methanol:ethanol (1:1, v/v)), mixed vigorously, and clarified by centrifugation (13,000 rpm for 15 min) then transferred to MS vials (33).

#### 2.4.2 Plant metabolite extraction

To extract metabolites from broccoli and alfalfa sprouts, sprouts were first freeze dried then extracted (30 mg freeze dried sprouts/700 μL ice cold methanol). Sprout and methanol mixture was homogenized on ice with Precellys beads then clarified with centrifugation (13,000 rpm for 10 min). Extracted supernatant from sprout samples was diluted 1:10 with 1:1 methanol:water (v/v) and transferred to MS vials. For collard greens, cooked collard green samples were homogenized using a hand-blender in an ice cold 80:20 (v/v) solution of methanol:water. Homogenate was centrifuged and extracted supernatant was diluted 1:10 with 80:20 (v/v) methanol:water.

### 2.5 Label identification approach summary

Our approach first uses HiResTEC to identify candidate metabolites from labeled or unlabeled samples. Next it extracts a set of features describing each isotopologue of a candidate metabolite and applies a random forest classifier to predict the probability that the isotopologue comes from a deuterated compound. Isotopologues are defined as a set of compounds which differ only in the number of isotopes they contain (here ^2^H atoms), and thus have the same structure and identity. For each candidate, we aggregate the predicted probabilities for all of its isotopologues from the labeled and unlabeled conditions, respectively. Lastly, using the aggregated probabilities the candidates are scored and the top scoring candidates are investigated for label incorporation.

### 2.6 Machine learning method for scoring candidates

The ML-based scoring of candidates consists of three main steps:

The first step applies a set of filtering rules to remove noisy candidate metabolites erroneously detected by XCMS. These filtering rules are created by hand, based on domain knowledge and manual inspection of typical noisy detections. The specific filtering rules are listed in Supplementary Table S2.

In the second step, we apply a classifier to predict the probability that each isotopologue comes from a labeled compound. As input to the classifier, each isotopologue is described by a set of features, which are described in Supplementary Table S3 (Figure 1C). In many isotopologues, not all four peaks are observed. The M_1_ peak is critical and most reliably available. Due to its critical importance, we impute the features associated with missing M_1_ peaks using a random forest classifier trained on complete isotopologue data. All features associated with M_2_ and M_3_ peaks are discretized with an additional “missing” category to address missing M_2_ and M_3_ peaks.

Our training data for building the classifier comes from metabolites extracted from the broccoli and alfalfa plants themselves. Labeled compounds in the plants display a high degree of label incorporation and HiResTEC has reasonable success in identifying labeled metabolites from these samples. While HiResTEC produces a reasonable set of true positive isotopologues, the resulting candidates do not resemble the typical isotopologues observed in human samples due to their high degree of labeling, thus not appropriate for training our classifier. To overcome this problem, labeled plant compounds rejected by HiResTEC were verified by human inspection to identify false negatives and build a training set that better resembles the isotopologue patterns anticipated to be observed in human samples. These candidates were selected by manually evaluating the 600 rejected candidates by HiResTEC. In total, our training data contains 600 candidates from broccoli and alfalfa. Some candidates were labeled only in broccoli or alfalfa, while others were labeled in both resulting in a total of 272 labeled and 928 unlabeled candidates.

We chose Random Forest (RF) as our classifier because it is particularly robust to overfitting, which is a crucial property as we need our classifier to apply to inputs that differ significantly from the training data. We tested the transferrability of the random forest classifier by training it using the alfalfa data and tested it on the broccoli data. It achieved an area-under-the-ROC-curve score of 0.95, and an area-under-the-precision-recall-curve score of 0.952 (see Supplementary Figure S1), indicating strong prediction performance and robustness to transfer.

Using the combined broccoli and alfalfa data set, we trained a Random Forest (RF) classifier with 100 trees (maximum depth = 5 and minimum instance counts =10) as implemented by the python package SciKitLearn (47). The hyperparameters were selected using cross-validation on the training data.

In the final step, we applied our trained RF model and produced a final score for each candidate (Figure 1D). Note that a candidate compound must be detected in multiple labeled samples as well as multiple unlabeled samples. We take all the isotopologues associated with a candidate and apply the RF classifier to predict the probability of each isotopologue being labeled. We then group all the isotopologues from the labeled samples and compute their average predicted probability of being labeled, denoted as p_L_. Similarly, we group all the isotopologues from the unlabeled samples and compute their average predicted probability of being labeled, denoted as p_U_. Finally, we compute the score of the candidate by $\frac{p_{L}}{p_{U}+c}$ , where p_U_ is used as a reference point to normalize p_L_. Here c = 0.01, and is introduced to ensure numerical stability. For a candidate to score high, its p_L_ must be significantly higher than its p_U_. This effectively allows us to rule out noisy detections that score high by our RF classifier but have similar p_L_ and p_U_ values.

To evaluate the generalizability of our approach, we tested it on two different biofluids derived from the consumption of 2 different cruciferous vegetables. First, we evaluated if the classifier, which was trained on plant-data, could provide useful ranking of the candidates for human urine data. We did a blinded test of three methods where candidates were generated using (1) our classifier-informed ranking method, (2) using *p*-values generated by HiResTEC, or (3) by randomly selecting candidates, and then evaluated for the plausibility of label being incorporated into the candidates. Our classifier-informed ranking method out-performed the other two methods indicating strong performance and usability. Given our success with this task, we next wanted to evaluate if our approach would work on plants that were significantly more labeled than the broccoli and alfalfa sprouts, so we used our approach on the urine from labeled-collard green consumers. Lastly, we wanted to evaluate our approach on another biofluid so we used our approach the plasma of labeled-collard green consumers.

### 2.7 Validation of deuterium incorporation

PeakView software (AB SCIEX, Framingham, MA) was used to validate the incorporation of label into metabolites selected by our machine learning model. Briefly, identified metabolites were searched across all samples using retention time and mass. The ratio of the intensity of two consecutive isotopologues ( $\frac{M_{n}}{M_{n+1}}$ ) was calculated for each compound and compared between the labeled and unlabeled conditions. Altered isotopologue ratios in the label-condition indicated the incorporation of deuterium into metabolites. Since we do not have a definitive chemical formula for most metabolites and we are not interested in the degree of label incorporation, we did not conduct natural isotope abundance correction.

### 2.8 Data and code availability

Metabolomics data is available on metabolomics workbench. Scripts and training data are available on github at “school-count/Metabolomics_project”.

## 3 Results

### 3.1 Hydroponic growth of plants in the presence of D_2_O labels metabolites

**Figure 2:**
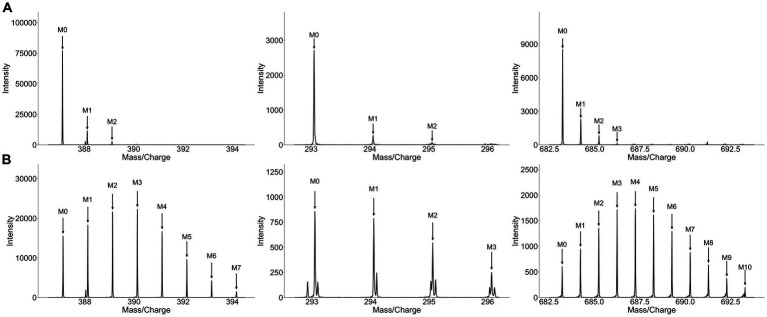
Growing sprouts in D_2_O significantly alters their isotopologue distribution. Spectra of matched unlabeled **(A)** and labeled **(B)** metabolites extracted from broccoli sprouts grown in the presence of H_2_O or 25% D_2_O, respectively. Each column represents one metabolite. Metabolites from broccoli grown in the presence of D_2_O display a markedly different isotopologue pattern exhibiting a greater number of isotopologues and an altered isotopologue ratio compared to metabolites from sprouts grown in H_2_O.

To validate that growing broccoli and alfalfa sprouts in the presence of D_2_O leads to deuterium incorporation and enrichment, plant material was analyzed using untargeted metabolomics. Subsequent analysis of the resulting MS data with HiResTEC led to many positive hits which were further hand validated for label incorporation (Figure 2). Overall, 195 metabolites were validated for label, 77 of which were labeled in both broccoli and alfalfa sprouts. Among the labeled metabolites that were unique to a single type of sprout, 56 of the labeled metabolites were labeled only in broccoli sprouts while 62 metabolites were labeled only in alfalfa sprouts. These results suggest unique compounds in each plant are labeled with deuterium and thus identification of label in human samples following consumption may be indicative of consumption of that vegetable. Analysis of the collard greens also showed a high level of label incorporation, with many positive hits using HiResTEC. In the collard green plants, a notably higher degree of deuterium incorporation was observed, presumably due to the longer exposure to label (3 months vs. 5 days). Overall, between both sprouts and collard greens, incorporation of label into plant material resulted in a greater number of isotopologues and an altered isotopologue ratio compared to the unlabeled controls (which contain naturally occurring ^13^C isotopes). Additionally, this analysis showed that deuterium-labeled metabolites can be detected via untargeted metabolomics without deuterium-hydrogen exchange occurring.

To evaluate the feasibility of using a machine learning approach to rank candidate labeled metabolites in human samples, a model was trained on candidates from the alfalfa sprouts and tested on candidates from the broccoli sprouts. The receiver operating characteristic (ROC) curve area under the curve (AUC) was 0.95 and the AUC of the precision-recall curve was 0.95 for predicting label (Supplementary Figure S1). These results indicated that our model was successful in discriminating between labeled and unlabeled metabolites in broccoli.

### 3.2 Labeled metabolites can be successfully detected in human urine and plasma

**Figure 3:**
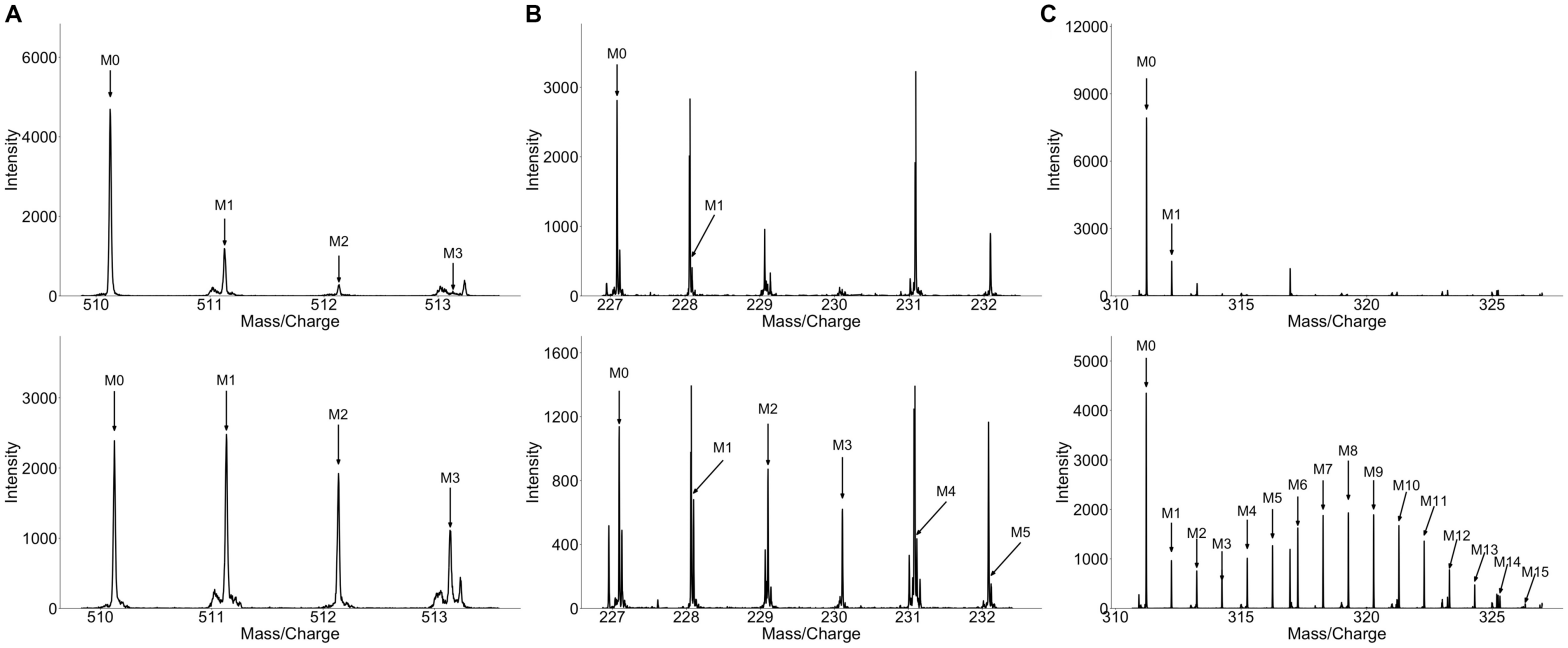
Spectra of metabolites recovered from the plasma and urine of broccoli sprouts and collard green consumers. Matched spectra from **(A)** broccoli sprout consumer urine, **(B)** collard green consumer urine, **(C)** collard green consumer plasma. Top panel represents unlabeled metabolites and bottom spectra represent matched labeled metabolites. The higher number of co-eluting peaks in **(B)** and high degree of label in **(C)** make detecting label particularly challenging.

Principal component analysis (PCA) revealed significant differences in metabolite profile of urine over time following broccoli sprout consumption (Supplementary Figure S2A). The 3 and 6 h time points were clearly separated on the PCA plots from baseline samples, while the 24 h collection was only modestly distinct. Importantly, samples for individuals who consumed labeled broccoli sprouts were not distinct from those consuming unlabeled sprouts at a given timepoint on the PCA plot. We next looked in the human samples for labeled metabolites and unlike in the plant material, HiResTEC software yielded hundreds of false positive and false negative candidate labeled metabolites. Sorting through these results was time and labor intensive. To address this issue, we used our machine learning model, which was trained on labeled and unlabeled metabolites from the vegetable samples, to prioritize which candidates to further investigate as labeled in human samples. We applied our model to metabolite data derived from the urine of subjects who consumed a single serving of broccoli sprouts grown in the presence of either D_2_O or H_2_O for 5 days. We successfully identified 6 metabolomic features representing 3 metabolites enriched with deuterium in the urine samples of individuals who consumed labeled broccoli (Figure 3A). Table 1 shows the mean isotopolgue ratio for each metabolite for all 16 labeled and unlabeled consumers as well as the standard deviation. Supplementary Table S4 shows the raw intensities of each isotoplogue for all samples measured and is organized by labeled metabolite (Supplementary Table S4). Supplementary Figure S3 provides the MS/MS data for labeled metabolites (Supplementary Figure S3). No labeled metabolites were detected in the urine of labeled nor unlabeled alfalfa sprout-consumers. The deuterium-enriched metabolites were only present in urine between 0–3 and 3–6 h. The fast metabolite excretion is consistent with a previous metabolomics study and indicated that these compounds are most likely bioactive xenobiotics (33). MS/MS matching to publicly available databases yielded no probable hits, thus we utilized *de novo* annotation techniques to predict class and molecular formula of identified metabolites (45). All three of the metabolites were predicted as glucuronidated compounds supporting the notion that the labeled metabolites were bioactive xenobiotics derived from broccoli which had been conjugated with glucuronic acid (Table 2). One of these metabolites we predicted to be indole-3-acetic acid-N-O-glucuronide supporting this hypothesis. Indeed, indole-3-acetic acid is a known plant hormone but it may also be derived from glucosinolates in the broccoli sprouts. The labeled metabolites were not present in urine of alfalfa sprouts consumers indicating that they are likely unique to broccoli (Supplementary Table S5).

**Table 1:** Mean isotopologue ratios of labeled-compounds detected in urine.

Study^a^	Ionization mode	RT	m/z	Time post consumption	Condition	M0/M1 ratio^b^	M1/M2 ratio	M2/M3 ratio	M3/M4 ratio
Broccoli Urine	Negative	23	426.1023	0–3 h	Unlabeled	4.71 ± 0.61	3.7 ± 1.7		
					Labeled	1.27 ± 0.16	1.68 ± 0.1		
				3–6 h	Unlabeled	4.04 ± 0.54			
					Labeled	1.2 ± 0.11	1.85 ± 0.13		
		21.18	366.0817	0–3 h	Unlabeled	4.61 ± 1.23	4.74 ± 1.3		
					Labeled	1.07 ± 0.12	1.96 ± 0.22		
				3–6 h	Unlabeled	4.28 ± 1.08	4.15 ± 1.0		
					Labeled	1.21 ± 0.11	1.91 ± 0.24		
		20.7	510.1234	0–3 h	Unlabeled	3.14 ± 1.0			
					Labeled	0.78 ± 0.16			
				3–6 h	Unlabeled	3.25 ± 1.0	4.27 ± 0.27	2.74 ± 1.6	
					Labeled	0.78 ± 0.12	1.31 ± 0.18	1.61 ± 0.18	
	Positive	23	404.0952	0–3 h	Unlabeled	4.01 ± 0.61	4.2 ± 0.64		
					Labeled	1.05 ± 0.10	1.83 ± 0.30		
				3–6 h	Unlabeled	3.69 ± 0.48			
					Labeled	1.18 ± 0.13	1.93 ± 0.24		
		21.18	390.0787	0–3 h	Unlabeled	3.84 ± 0.81	3.85 ± 0.67		
					Labeled	1.0 ± 0.17	2.12 ± 0.22		
				3–6 h	Unlabeled	1.03 ± 0.55			
					Labeled	0.77 ± 0.28			
		20.7	512.1394	0–3 h	Unlabeled	2.85 ± 1	3.67 ± 0.85		
					Labeled	0.72 ± 0.13	1.50 ± 0.16		
				3–6 h	Unlabeled	2.78 ± 0.91	3.51 ± 1.1		
					Labeled	0.70 ± 0.11	1.41 ± 0.16		
Collard Greens Urine	Negative	17.79	250.0728	0–24 h	Unlabeled	7.70 ± 0.41			
					Labeled	4.06 ± 0.71	1.95 ± 0.75	1.86 ± 0.40	2.67 ± 0.30
		15.91	251.0138	0–24 h	Unlabeled	8.77 ± 0.34			
					Labeled	0.88 ± 0.44	0.99 ± 0.07	2.16 ± 0.34	
		18.97	289.0394	0–24 h	Unlabeled	7.54 ± 0.21			
					Labeled	4.12 ± 0.9	1.54 ± 0.33	1.36 ± 0.1	1.49 ± 0.34
		20	225.0765	0–24 h	Unlabeled	9.68 ± 1.69	4.18 ± 0.95		
					Labeled	4.51 ± 2.04	0.57 ± 0.16	1.81 ± 0.95	1.26 ± 0.11
	Positive	20	227.0907	0–24 h	Unlabeled	7.5 ± 1.13			
					Labeled	2.52 ± 1.0	0.75 ± 0.17	1.66 ± 0.57	1.93 ± 0.65
		20	249.0727	0–24 h	Unlabeled	8.85 ± 1.37			
					Labeled	2.75 ± 1.11	0.63 ± 0.17	1.82 ± 0.74	1.55 ± 0.21
		21.17	304.2494	0–24 h	Unlabeled	9.07 ± 1.42			
					Labeled	2.14 ± 0.93	1.22 ± 0.33	1.3 ± 0.23	1.76 ± 0.37

a Data are from participants that consumed either labeled broccoli sprouts (*n* = 8), unlabeled broccoli sprouts (*n* = 8), or labeled collard greens (*n* = 21) as indicated in the table.

b Data are the mean ratio of the indicated isotope abundance ± standard deviation in the indicated ratio.

**Table 2:** Masses, retention times, and annotations of labeled-compounds.

Study	Retention time	m/z	Adduct	Neutral mass	Identification
Broccoli Urine	23	426.1023	[M-H + FA]-	381.105	*Predicted: Glucuronidated Compound*
		404.0952	[M + Na]+		
	21.18	366.0817	[M-H]-	367.0892	*Predicted: indole-3-acetic acid-N-O-glucuronide*
		390.0787	[M + Na]+		
	20.7	510.1234	[M-H]-	511.1314	*Predicted: Glucuronidated Compound*
		512.1394	[M + H]+		
Collard Greens Urine	20	225.0765	[M-H]-	226.0835	*Predicted: Dihydrosinapic Acid*
		227.0907	[M + H]+		
		249.0727	[M + Na]+		
	21.17	304.2494	[M +?]+		*Predicted:* No MS/MS Captured
	17.79	250.0728	[M +?]-		*Predicted: N-Acyl-Alpha Amino Acids*
	15.91	251.0138	[M +?]-		*Predicted: Aminopyrimidines*
	18.97	289.0394	[M +?]-		*Predicted: Sulfuric Acid Monoesters*
Collard Greens Plasma	6.36	130.0867	[M-H]+	131.0939	Isoleucine
		132.1011	[M + H]+		
	24.8	311.2229	[M-H]+	312.2291	*Predicted: Linoleic Acids and Derivatives*
		295.2247	[M + H-H2O]+		
	24.8	313.2372	[M-H]+	314.2426	No MS/MS Captured
		297.2375	[M + H-H2O]+		
	24.8	333.2044	[M-H]-	334.2113	*Predicted: Alkaloids*
		335.2181	[M + H]+		
	24.8	335.2199	[M-H]-	336.2254	No MS/MS Captured
		403.2071	[M-H + HCOONa]-		
		337.2327	[M + H]+		

As our model was successful in identifying labeled metabolites from the urine of individuals who consumed labeled broccoli sprouts, we wanted to evaluate the model’s ability to identify labeled metabolites from other vegetables. We next applied our model to the metabolite data derived from urine of individuals who consumed collard greens grown in the presence of 33% D_2_O for 3 months. PCA showed modest differences in the urine between the baseline and 24 h post collards sample (Supplementary Figure S2B) which may be due to a difference in urine collection method (total 24 h urine collection vs. 0–3, 3–6 time frame windows). In the urine of collard greens consumers, we detected 7 metabolomic features representing 5 metabolites which were enriched with deuterium (Table 1; Figure 3B). Of the detected metabolites, one was predicted to be dihydrosinapic acid, an *N*-acyl-alpha amino acid, an aminopyrimidine, and a sulfuric acid monoester (Table 2). All labeled compounds detected in the collard green consumers’ urine except the aminopyrimidine were found to be present, but not labeled, at all timepoints in both the alfalfa and the broccoli consumers’ urine (Supplementary Table S5). Conversely, the aminopyrimidine was found in neither the alfalfa nor broccoli consumers’ urine. Comparison of the presence of labeled metabolites across treatment groups is shown in Supplementary Table S5.

To evaluate the usability of our approach on other biological fluids, and compare signatures discovered in plasma to those in urine, we applied our model to metabolomics data generated from plasma samples collected 4 h following the consumption of labeled collard greens. In the plasma, we detected deuterium-incorporation in 11 metabolomic features corresponding to 5 metabolites. Supplementary Table S6 shows the mean isotopolgue ratio for each metabolite for all 16 labeled and unlabeled consumers as well as the standard deviation. Of these 5 metabolites, one was annotated as isoleucine via our in-house library, one was predicted to be a linoleic acid or derivative, one was predicted as an alkaloid, and the final two did not have MS/MS information (Table 2). The detected fatty acid has a markedly different isotopologue pattern compared to the other detected deuterium-labeled metabolites and the fatty acid’s isotopologue pattern was more similar to those detected in the plants themselves as opposed to those detected in urine (Figure 3C).

Taken together, these results indicate that deuterium-labeled metabolites can be recovered from human plasma and urine following the consumption of foods intrinsically labeled with deuterium. Furthermore, our ML-based approach to label identification is fast, flexible and yields positive results in both human urine and plasma samples as well as from samples collected after the consumption of two different plants grown in the presence of deuterium for different lengths of time.

## 4 Discussion

In this study, participants were fed broccoli sprouts, alfalfa sprouts, and collard greens grown in the presence of D_2_O to label their metabolites. First, we showed that plant metabolites can be successfully enriched with deuterium, intrinsically labeling their metabolites. This approach was taken to identify food-derived biosignatures of cruciferous vegetable consumption as opposed to endogenous compounds which are altered with consumption, or effect biomarkers. Next, we met our objective of conducting global untargeted stable isotope tracing and demonstrated that a machine learning classifier trained on data generated from deuterium-labeled plants can be used to prioritize metabolites in human urine and plasma for discovery of labeled human metabolites that may act as biosignatures of food consumption. These compounds can potentially be used for further annotation and validation as food biomarkers. While not an automatic process, our machine learning approach allowed us to quickly sort through thousands of metabolites for those most-likely to be labeled. This helped resolve the challenges presented by currently available software which yields a high number of false positives and false negatives. Using our machine learning approach, we successfully identified a total of 24 deuterium-labeled metabolomic features in human urine and plasma which corresponded to 8 metabolites in urine and 5 metabolites in plasma. The presence of label in these metabolites allows us to conclude that the metabolites were directly derived from the food of interest, and not an endogenous metabolite that increased in abundance in response to the consumption of the study food. These metabolites may represent novel biosignatures of broccoli sprout consumption in our clinical trials, however, validation of these metabolites as biosignatures will require more research across a larger and more diverse cohort of individuals. Additionally, their rapid elimination suggest they are bioactive. To our knowledge, this is the first time global untargeted stable isotope traced metabolomics has been successfully used in human samples to identify labeled metabolites from a food-source. Importantly, as global untargeted stable isotope traced metabolomics has been used broadly across the life sciences, our new research tool published here should be useful to other fields of research (14, 48, 49).

In recent years there has been increasing interest in identifying biological signature of foods as biomarkers of food intake (6–9). This study represents a significant advance in this area and presents a novel framework and approach for biomarker discovery. While food-metabolite based biomarker discovery has typically been carried out using untargeted metabolomics without labeled foods, these analyses cannot separate between host- and plant-derived metabolites (11, 12, 50). Food derived-compounds which are not present before consumption generate superior biomarker candidates compared to host-derived metabolites, thus presenting a key advantage of our work (7). Previous work conducted in rodents has proposed circulating glutathione levels as a potential biomarker of cruciferous vegetable consumption (11). While glutathione may serve as an “effect biomarker,” glutathione levels are known to be influenced by other dietary components such as polyphenols and vitamin E making it a poor food intake biomarker for cruciferous vegetables (7, 51–53). Discovering biomarkers for food groups that are specific to the food group is a key challenge that research in this field faces. We identified only a small number of labeled-metabolites in the urine of broccoli-consumers and these metabolites were present for a limited period of time (3–6 h post consumption). However, some of these metabolites represent compounds which appear to be unique to broccoli. The fast excretion of these compounds follows a similar pattern to sulforaphane and other isothiocyanates, compounds which are unique to cruciferous vegetables and which are known for their anti-cancer effects (31, 32, 35, 36). Recent work employing machine learning to identify biomarkers of broccoli consumption in the fecal metabolome exhibited poor performance, most likely due to a high overlap between compounds from broccoli and those from other dietary sources (50). These findings may also explain why no labeled metabolites were detected in people who consumed alfalfa sprouts: there were no dietary restrictions on other plants from family Fabaceae, thus any unique alfalfa compounds were diluted by other dietary sources. Future work is needed to validate the specificity and use of the detected-labeled compounds as signatures and biomarkers of broccoli sprout consumption. Additionally, work is also needed to determine whether the labeled compounds we detected are present following consumption of mature broccoli, akin to what consumers commonly purchase at the supermarket. Another avenue of important future work is the elucidation of the structure, and investigation of the potential bioactivity of the identified labeled compounds.

**Figure 4:**
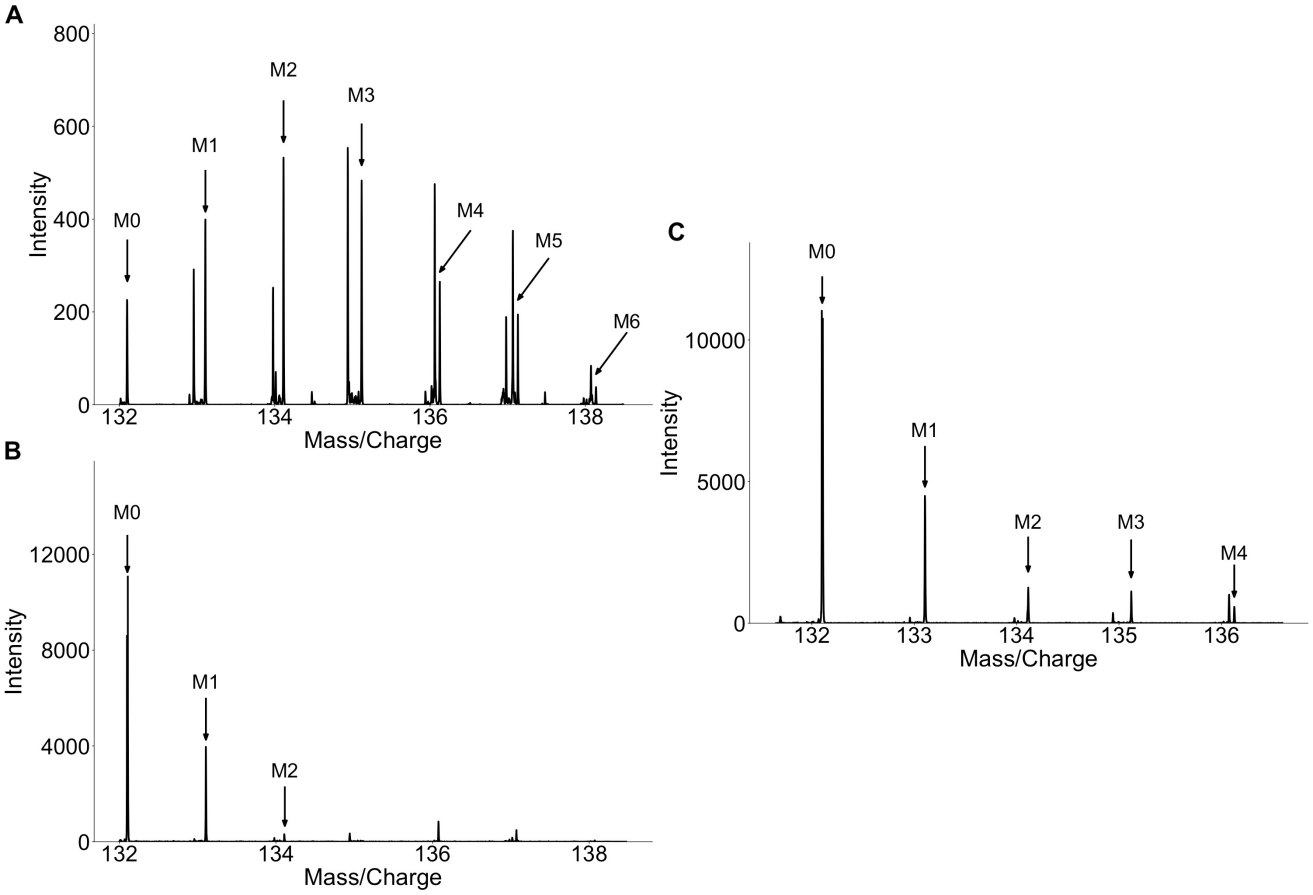
Spectra of isoleucine from **(A)** collard greens, **(B)** baseline plasma, **(C)** 4 h plasma. Isoleucine detected in collard green plant show a high degree of label **(A)** while isoleucine detected in circulation prior to consumption of labeled collard greens contains no label **(B)**. Isoleucine detected in circulation 4 h after the consumption of labeled collard greens shows a similar M_0_ and M_1_ pattern as at baseline, however, M_2_, M_3_, and M_4_ can be detected representing the deuterium-incorporated isotopologues.

The discovery of isoleucine, indole-3-acetic acid-N-O-glucuronide, and dihydrosinapic acid as labeled metabolite from collard greens or broccoli sprouts highlights the difficulties in identifying food specific biosignatures and biomarkers. Due to its lack of specificity to cruciferous vegetables these metabolites are poor biomarkers. These metabolites are an example of a major limitation to the methodology, namely the potential biomarkers identified may not be specific to the food source as other food groups may also contain the same metabolite. In the case of isoleucine it was enriched with deuterium because of the long duration of collard greens being grown with D_2_O. While one would assume that the label pattern of isoleucine from collard greens would be diluted due to the high abundance of isoleucine, both in circulation and from other dietary components, investigation of the label patterns at baseline, in the plants, and following consumption of labeled sprouts gives greater insight into the disposition of label *in vivo*. In the plant, many isotopologues are observed (Figure 4A), while at baseline (pre-consumption of plants) only the monoisotopic mass (M_0_) and an isotopologue containing a singular ^13^C atom (M_1_) are observed (Figure 4B). In plasma, however, while the ratio of M_0_ and M_1_ look similar in between the labeled- and unlabeled- collard green consumers, the labeled-consumers exhibit a tail of heavier isotopologues (M_2_, M_3_, M_4_) which are not present in the unlabeled-consumers (Figure 4C). This heavy isotopologue tail can be assumed to be directly attributed to the presence of these heavier isotopologues in the plant, thus, isotopologue distribution detected in circulation appears to be a mixture of the isotopologue patterns observed at baseline and in the plants. Similarly, other metabolites detected in the urine of collard green consumers appeared in the urine of broccoli and alfalfa consumers, including at baseline. This again highlights the difficulty of identifying specific biomarkers of food as some compounds are most likely non-specific plant compounds that were labeled due to the long growing period of collard greens with D_2_O. Future work should consider the degree of label achieved in the food of interest to work towards discovering biomarkers that are specific to the food of interest.

A major limitation of this study is the lack of annotations for many of the deuterium-labeled metabolites we identified which is a problem with food biomarker discovery. Indeed, we tentatively annotated a small number of metabolites however these compounds are common and well known plant metabolite that lack specificity to cruciferous vegetables. Within the field of metabolomics, annotation of metabolites is typically completed via MS/MS matching using databases, however, these databases are typically skewed towards endogenous compounds and pharmaceuticals while lacking plant- and bacterial-derived metabolites (54–56). Given that the deuterium-labeled metabolites are derived directly from the plants, it is unsurprising that MS/MS matching yielded poor results. To overcome this problem, a *de novo* annotation software was utilized to broadly predict the classes of the detected metabolites, which while informative is less specific and has higher uncertainty than MS/MS matching. Unsurprisingly, some of the predicted classes were for compounds that contained glucuronides, which are conjugated with glucuronic acid. These findings support our hypothesis that many of the labeled metabolites we identified are bioactive secondary plant metabolites as a major excretory pathway for xenobiotics is via glucuronic acid conjugation. While these findings support our hypothesis, the metabolism and biotransformation of these metabolites creates a further challenge in annotation as the parent metabolite in the plants may not have a similar mass nor retention time. Additionally, for compounds such as glucosinolates, which undergo enzymatic hydrolysis to become bioactive, many plant derived metabolites may have different structures altogether then the parent compounds in plants (57, 58). Other limitations in the plant labeling and LC–MS/MS methodology should also be considered for future work in this field. We did not detect any labeled isothiocyanates, presumably because the glucosinolates in the sprouts were already formed in the seeds of the plants and thus glucoraphanin did not incorporate label. Our chromatographic method is focused on polar compounds (i.e., metabolites), thus chromatographic resolution for highly non-polar compounds, such as fatty acids, is limited. Previous work using deuterated collard greens have shown that both vitamins E and K become deuterated, however, due to their strong lipophilicity we could not detect them in our analysis (30, 59–62). Likewise, we did not identify a potential biomarker of cruciferous vegetable consumption called S-methyl-L-cysteine sulfoxide (SMCSO) in our LC–MS/MS data due to limitations in LC–MS/MS methodology (63).

A major innovation of our study was the application of machine learning for biological signature discovery in conjunction with stable-isotope tracing. Currently available software tools perform poorly on metabolites with a low degree of labeling, such as those in human urine, yielding a high number of false positives and false negatives. A key challenge we faced was the lack of training examples from the human samples. Instead, we had to resort to training our model from plant data, for which the labeled metabolites have a higher degree of deuterium-incorporation and are easier to detect. Our approach took a model learned on such “clean” data and successfully adapted it for human data. Data generated from this study, and others using similar approaches, can be compiled to generate new training data which is more robust and representative of metabolites in human samples to supplement this data.

In conclusion, in this study we utilized a machine-learning approach to rapidly prioritize candidate metabolites to evaluate for label incorporation. We applied this approach to untargeted metabolomics data of human urine and plasma following the consumption of deuterium-labeled vegetables to identify biological signatures of cruciferous vegetable consumption. A major strength of our approach is that it allows us to identify signatures of food intake derived directly from broccoli sprouts or collard greens as opposed to host metabolites which reflect a functional response to a food exposure. This work highlights an innovative use of machine learning in biological sciences, a field likely to grow in the coming future. All in all, this work presents a novel approach to identifying biological signatures of food consumption for biomarker discovery and proves the feasibility of global untargeted stable isotope tracing in humans. Similar approaches can be applied to other foods and environmental exposures potentially advancing knowledge and accelerating signature and biomarker discovery in fields such as nutrition, environmental and molecular toxicology, and the pharmaceutical sciences.

## Data availability statement

The datasets presented in this study can be found in online repositories. The names of the repository/repositories and accession number(s) can be found below: metabolomics data is available on metabolomics workbench (https://www.metabolomicsworkbench.org/). Scripts and training data are available on github at “school-count/Metabolomics_project”.

## Ethics statement

The studies involving humans were approved by Oregon State University Institutional Review Board (IRB-2019-0123, IRB8343) and Tufts University Institutional Review Board (IRB7421). The studies were conducted in accordance with the local legislation and institutional requirements. The participants provided their written informed consent to participate in this study.

## Author contributions

JB: Conceptualization, Data curation, Formal analysis, Investigation, Validation, Writing – original draft, Writing – review & editing, Methodology. YR: Writing – original draft, Formal analysis, Investigation, Methodology. LB: Investigation, Writing – original draft, Writing – review & editing. JC: Investigation, Writing – review & editing. CW: Writing – review & editing, Investigation. LH: Investigation, Writing – review & editing. MT: Supervision, Writing – original draft, Writing – review & editing. JK: Investigation, Writing – original draft. SB: Writing – original draft, Writing – review & editing, Supervision. JS: Conceptualization, Funding acquisition, Supervision, Writing – original draft, Writing – review & editing. XF: Funding acquisition, Writing – original draft, Writing – review & editing, Supervision, Investigation. EH: Conceptualization, Funding acquisition, Supervision, Writing – original draft, Writing – review & editing.
