# Supplementary material for: Identification of biological signatures of cruciferous vegetable consumption utilizing machine learning-based global untargeted stable isotope traced metabolomics
Source: Front Nutr. 2024 Jul 3;11:1390223. doi: 10.3389/fnut.2024.1390223 (PMC11253721; doi:10.3389/fnut.2024.1390223)
Supplement: Supplementary file 7 [file Presentation_1.pptx]

## Slide 1
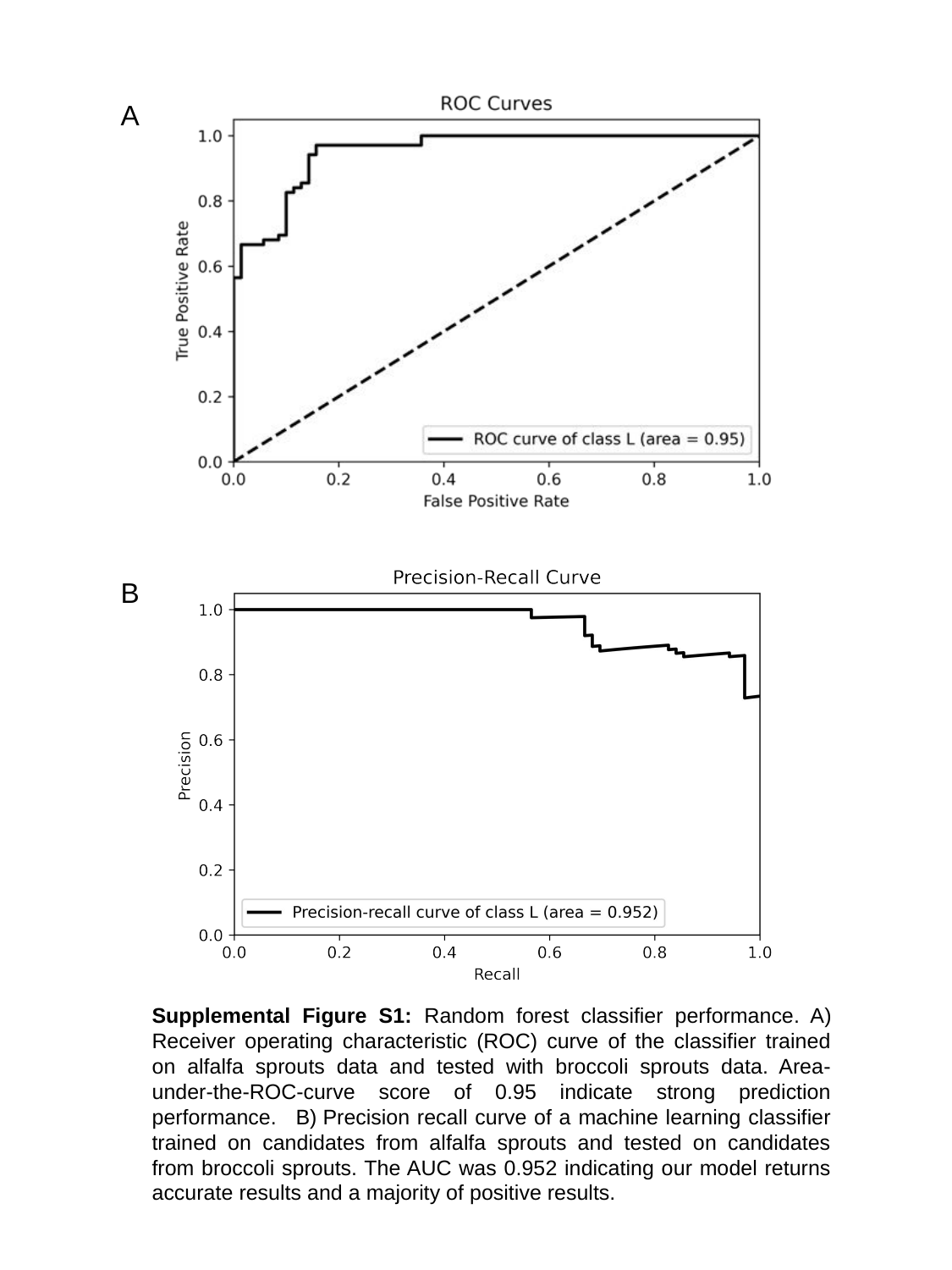

A
B
Supplemental Figure S1: Random forest classifier performance. A) Receiver operating characteristic (ROC) curve of the classifier trained on alfalfa sprouts data and tested with broccoli sprouts data. Area-under-the-ROC-curve score of 0.95 indicate strong prediction performance.   B) Precision recall curve of a machine learning classifier trained on candidates from alfalfa sprouts and tested on candidates from broccoli sprouts. The AUC was 0.952 indicating our model returns accurate results and a majority of positive results.
