# Supplementary material for: Identification of biological signatures of cruciferous vegetable consumption utilizing machine learning-based global untargeted stable isotope traced metabolomics
Source: Front Nutr. 2024 Jul 3;11:1390223. doi: 10.3389/fnut.2024.1390223 (PMC11253721; doi:10.3389/fnut.2024.1390223)
Supplement: Supplementary file 8 [file Presentation_2.PPTX]

## Slide 1
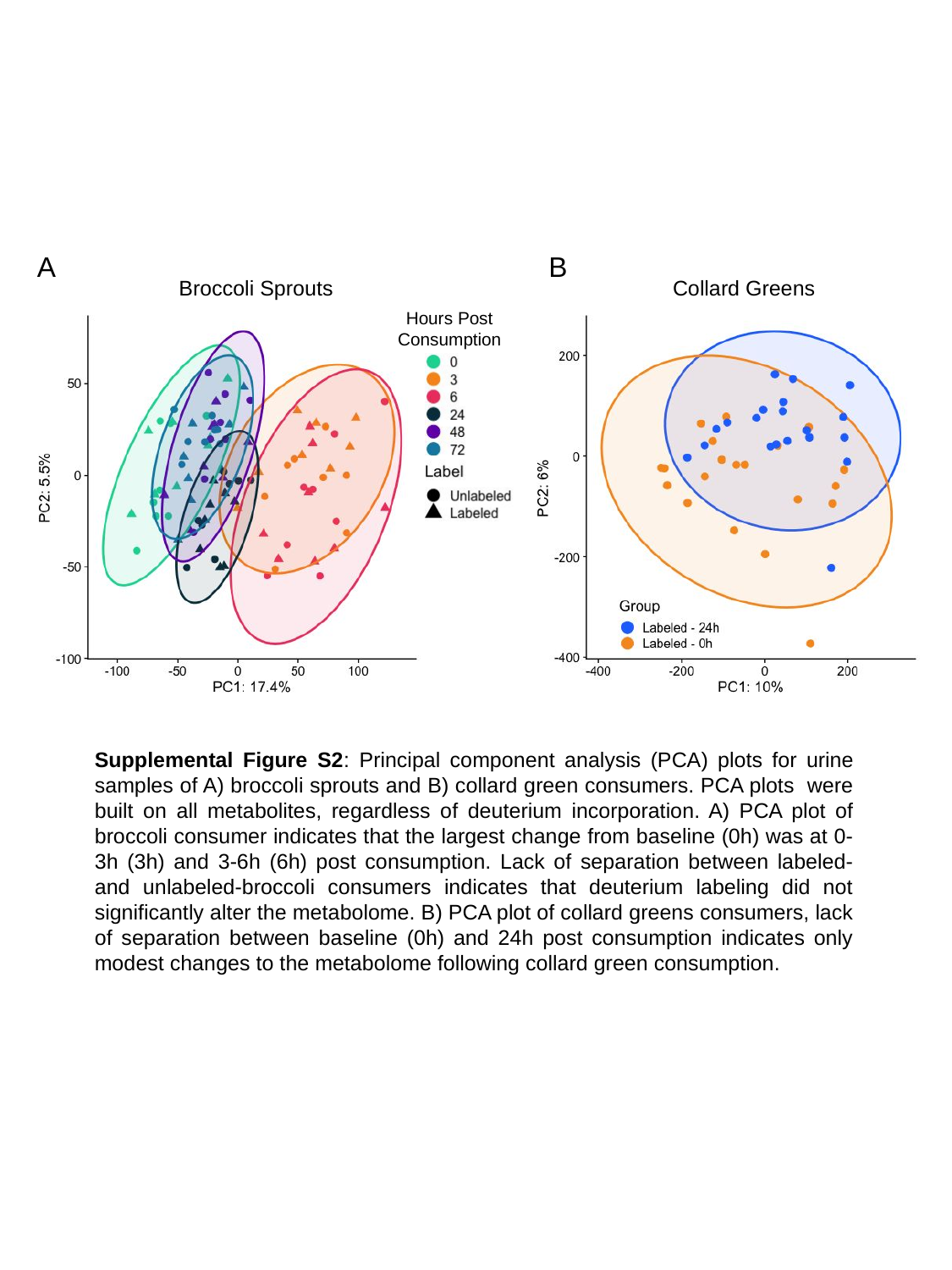

A
B
Broccoli Sprouts
Collard Greens
Hours Post
Consumption
Supplemental Figure S2: Principal component analysis (PCA) plots for urine samples of A) broccoli sprouts and B) collard green consumers. PCA plots were built on all metabolites, regardless of deuterium incorporation. A) PCA plot of broccoli consumer indicates that the largest change from baseline (0h) was at 0-3h (3h) and 3-6h (6h) post consumption. Lack of separation between labeled- and unlabeled-broccoli consumers indicates that deuterium labeling did not significantly alter the metabolome. B) PCA plot of collard greens consumers, lack of separation between baseline (0h) and 24h post consumption indicates only modest changes to the metabolome following collard green consumption.
