# Supplementary material for: Identification of biological signatures of cruciferous vegetable consumption utilizing machine learning-based global untargeted stable isotope traced metabolomics
Source: Front Nutr. 2024 Jul 3;11:1390223. doi: 10.3389/fnut.2024.1390223 (PMC11253721; doi:10.3389/fnut.2024.1390223)
Supplement: Supplementary file 9 [file Presentation_3.PPTX]

## Slide 1
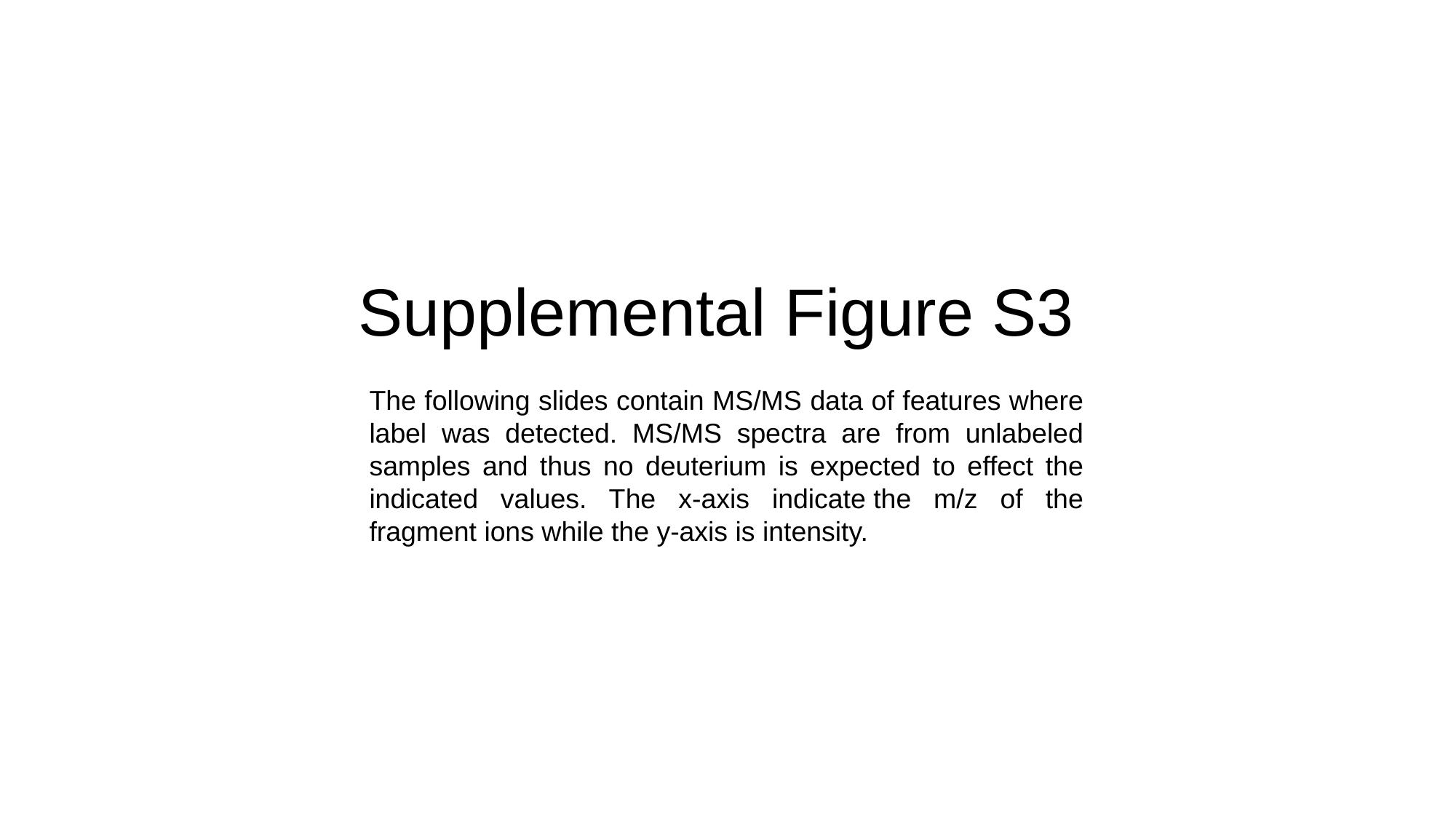

# Supplemental Figure S3
The following slides contain MS/MS data of features where label was detected. MS/MS spectra are from unlabeled samples and thus no deuterium is expected to effect the indicated values. The x-axis indicate the m/z of the fragment ions while the y-axis is intensity.

## Slide 2
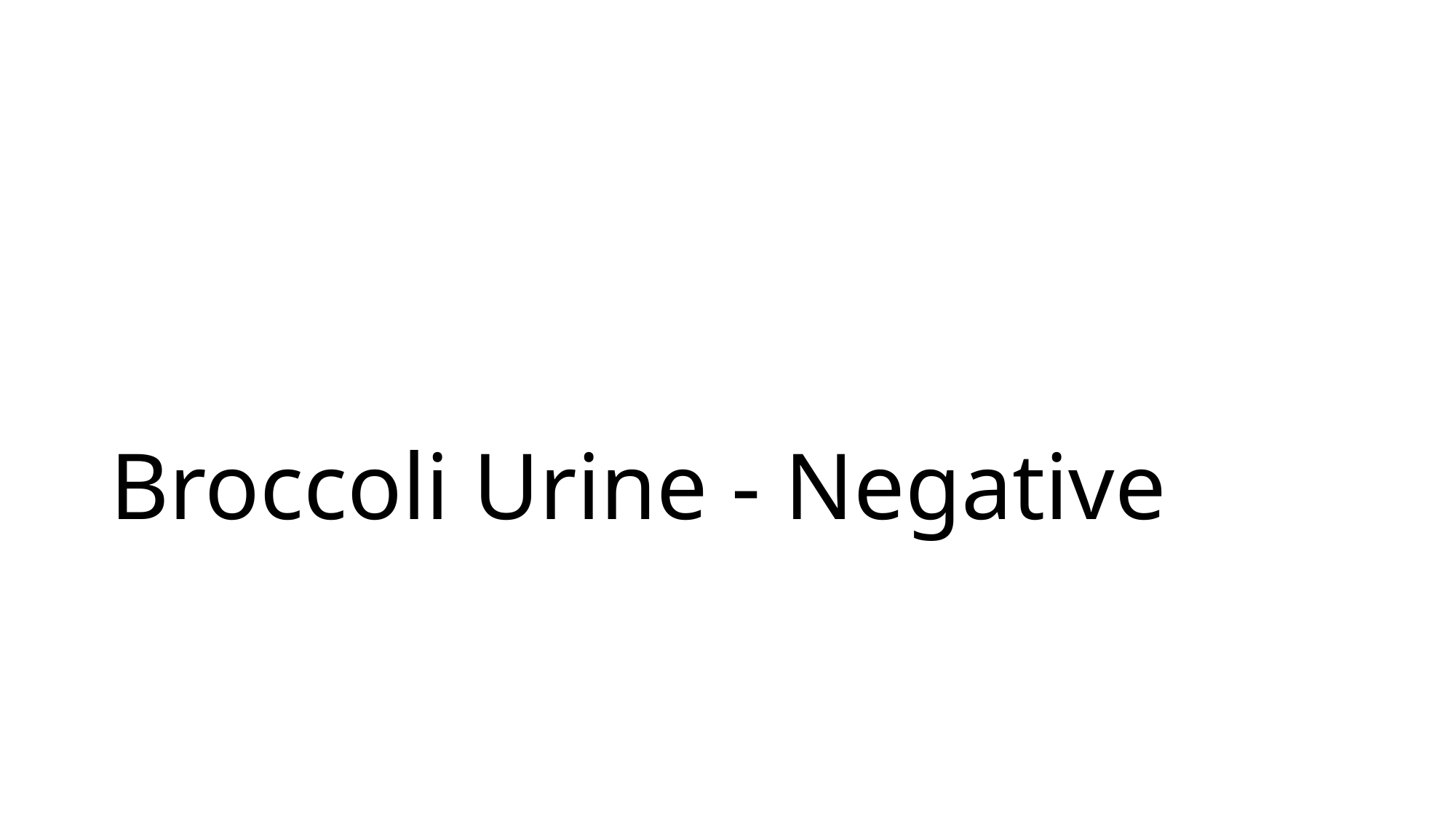

# Broccoli Urine - Negative

## Slide 3
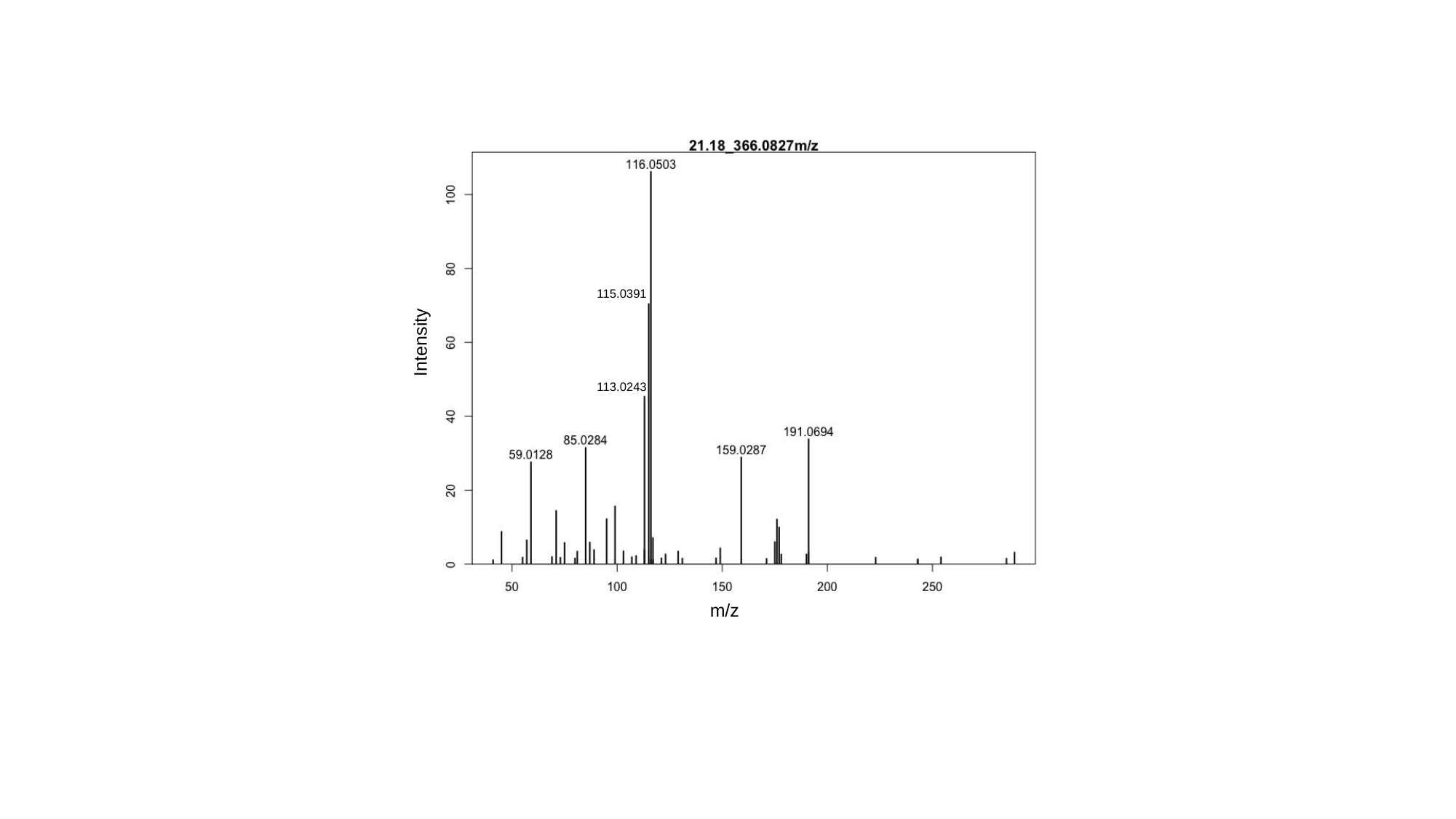

115.0391
113.0243
Intensity
m/z

## Slide 4
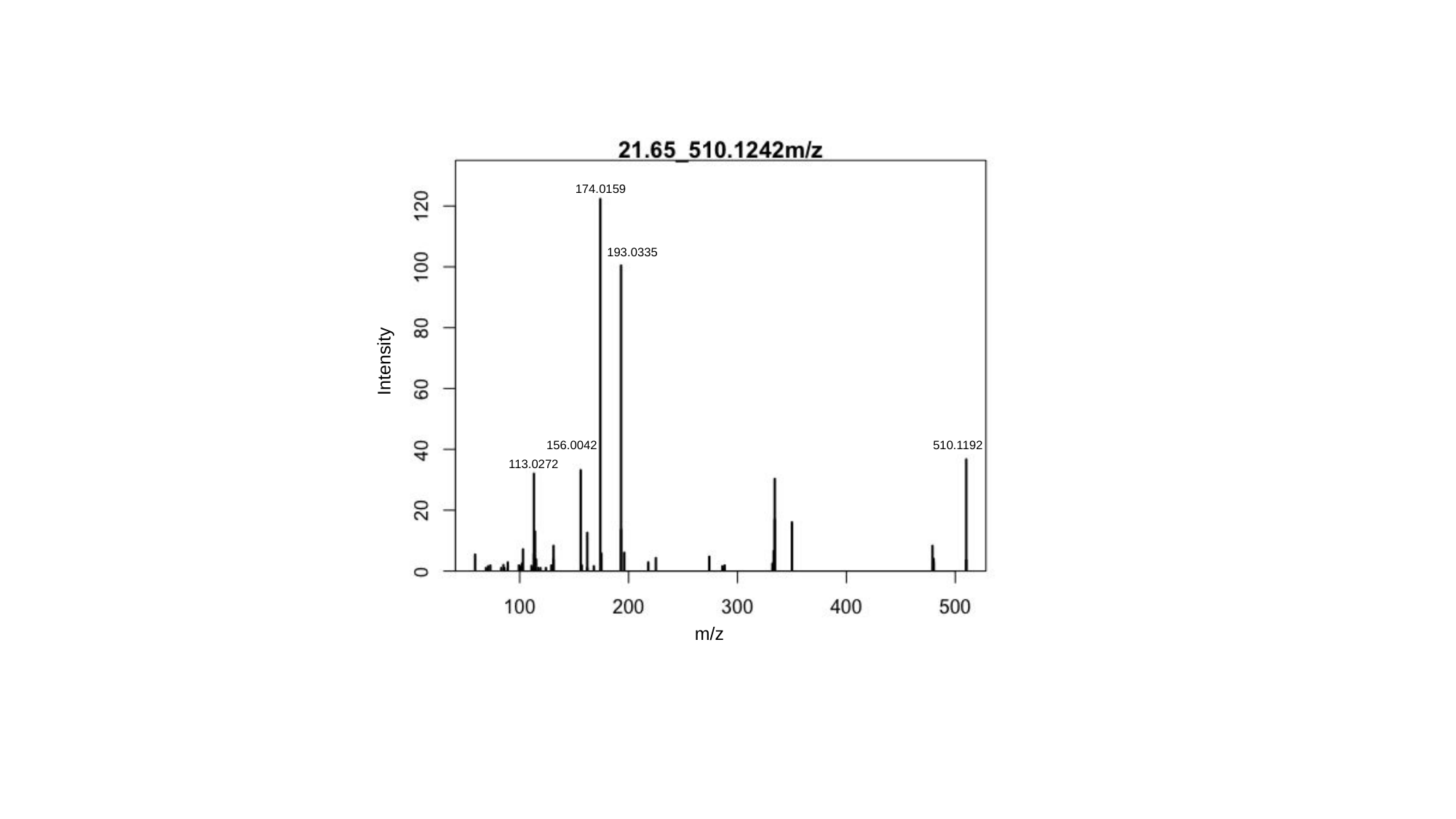

174.0159
193.0335
156.0042
510.1192
113.0272
Intensity
m/z

## Slide 5
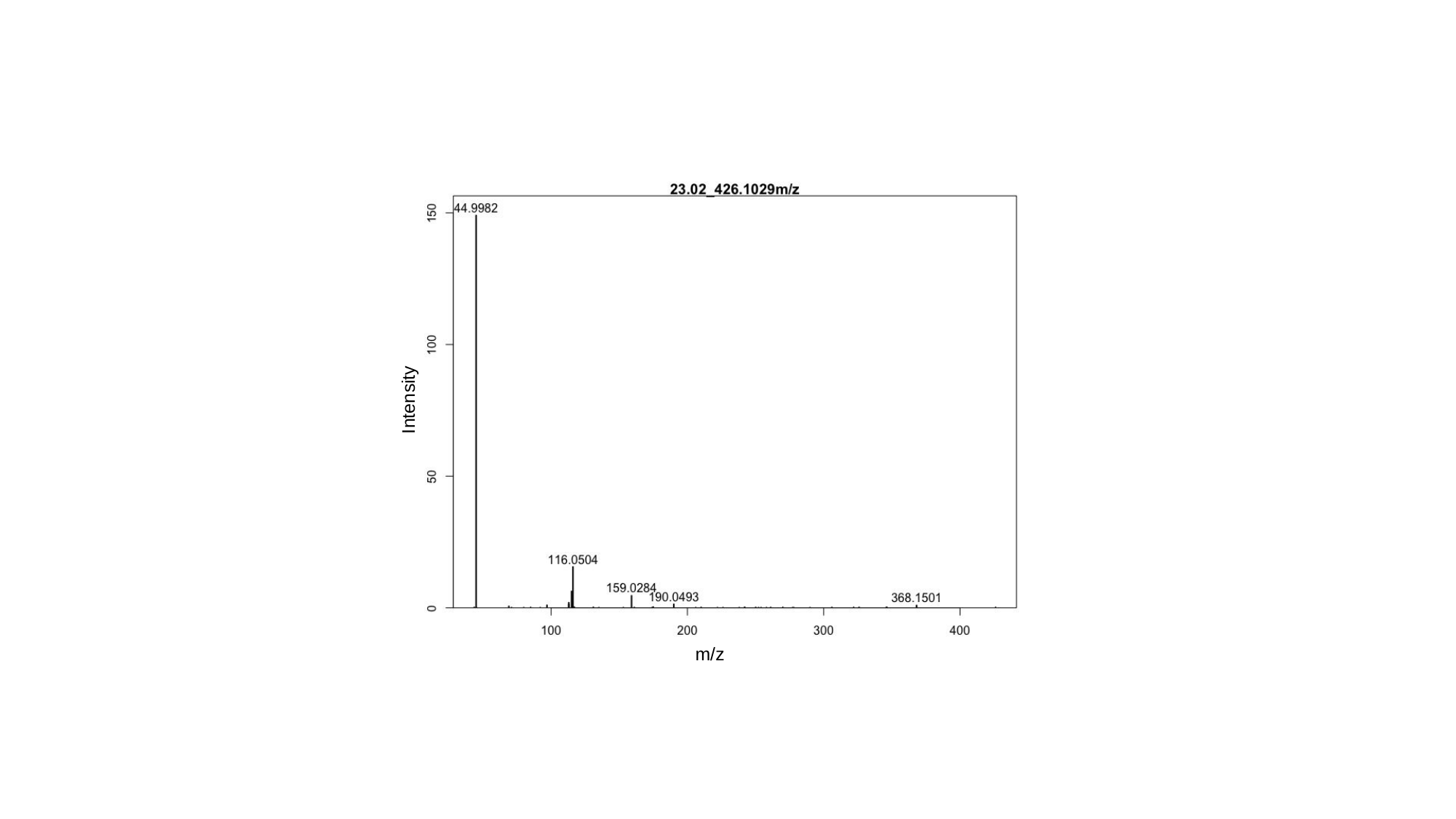

Intensity
m/z

## Slide 6
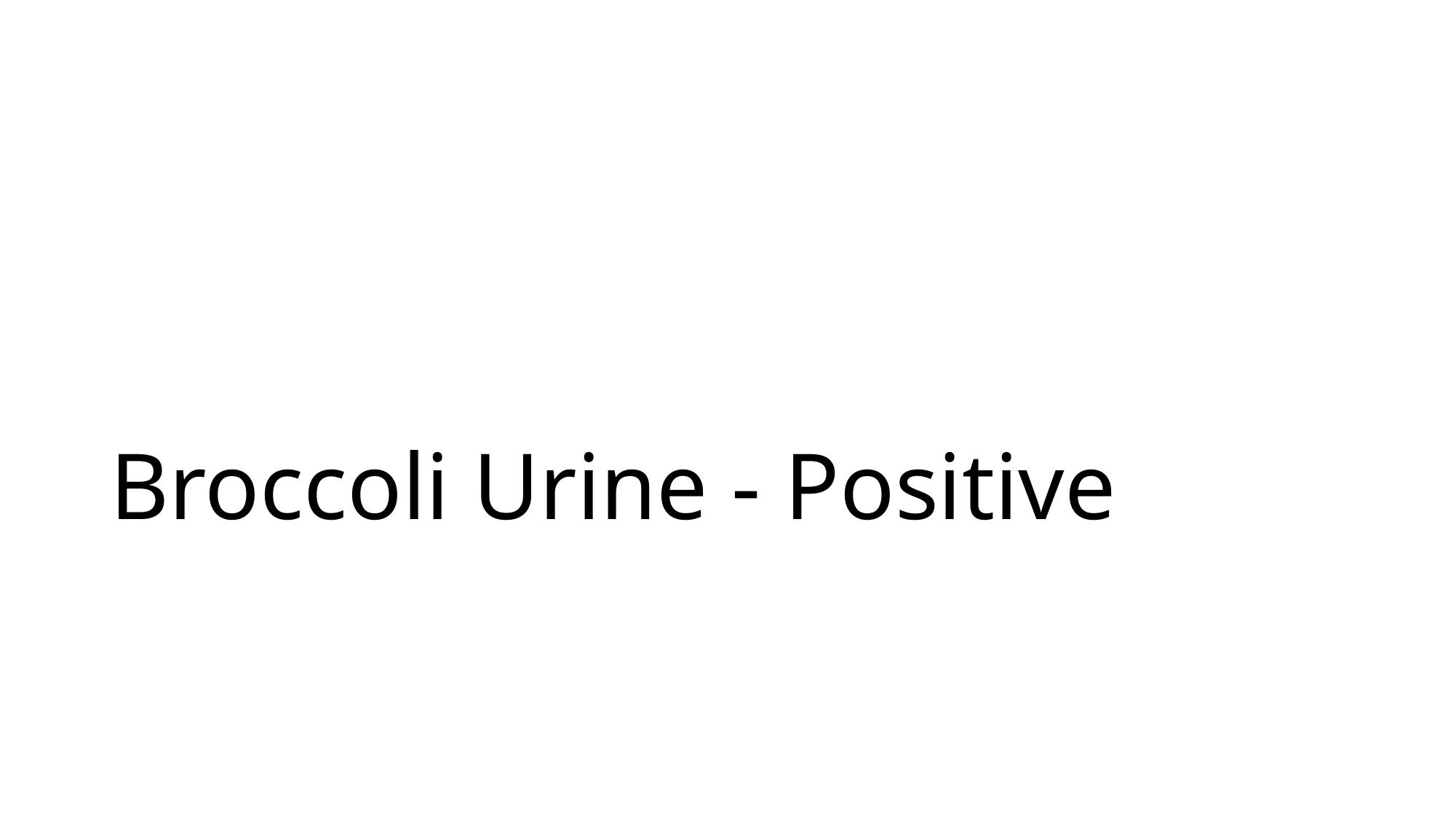

# Broccoli Urine - Positive

## Slide 7
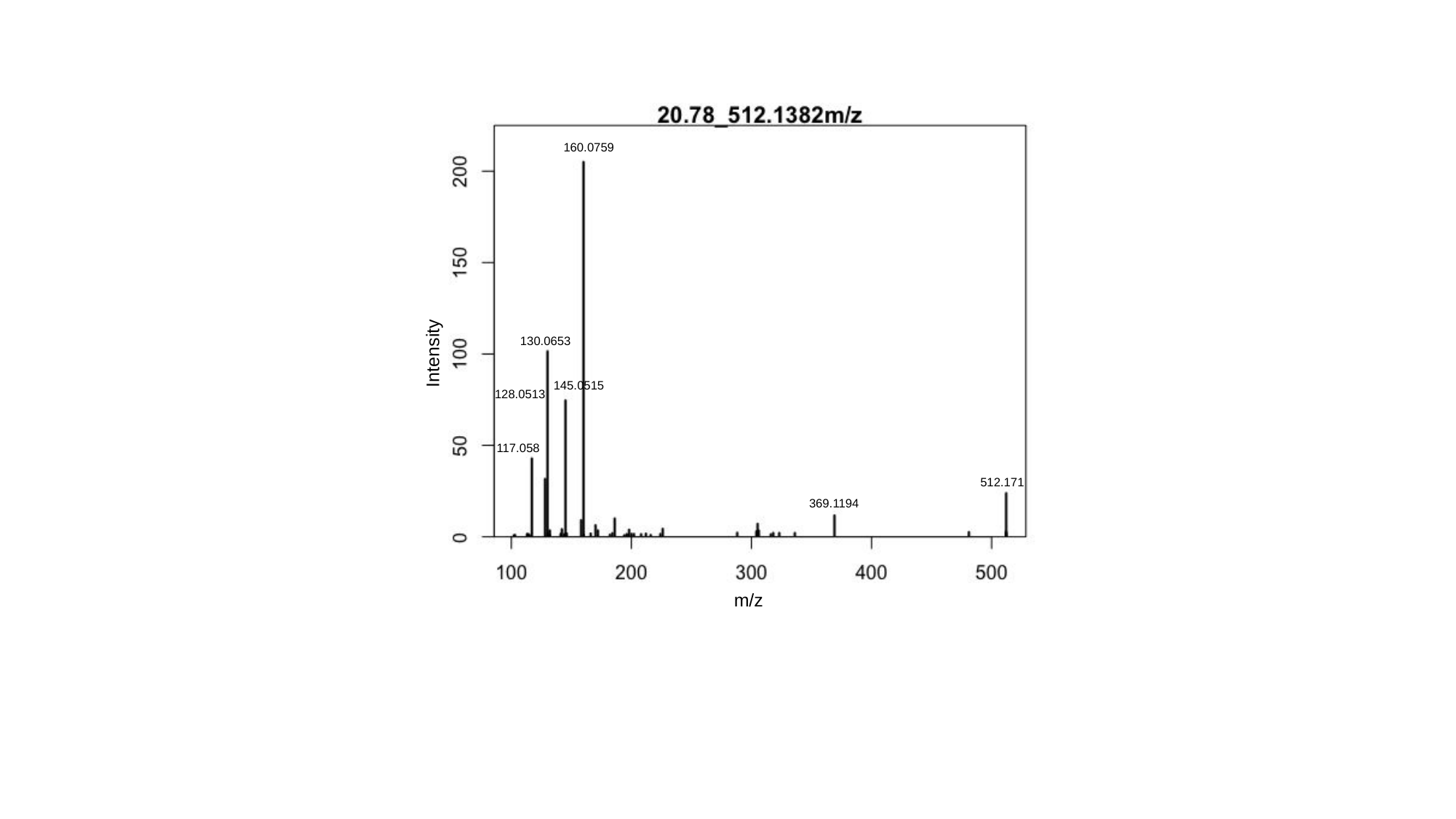

160.0759
130.0653
145.0515
128.0513
117.058
512.171
369.1194
Intensity
m/z

## Slide 8
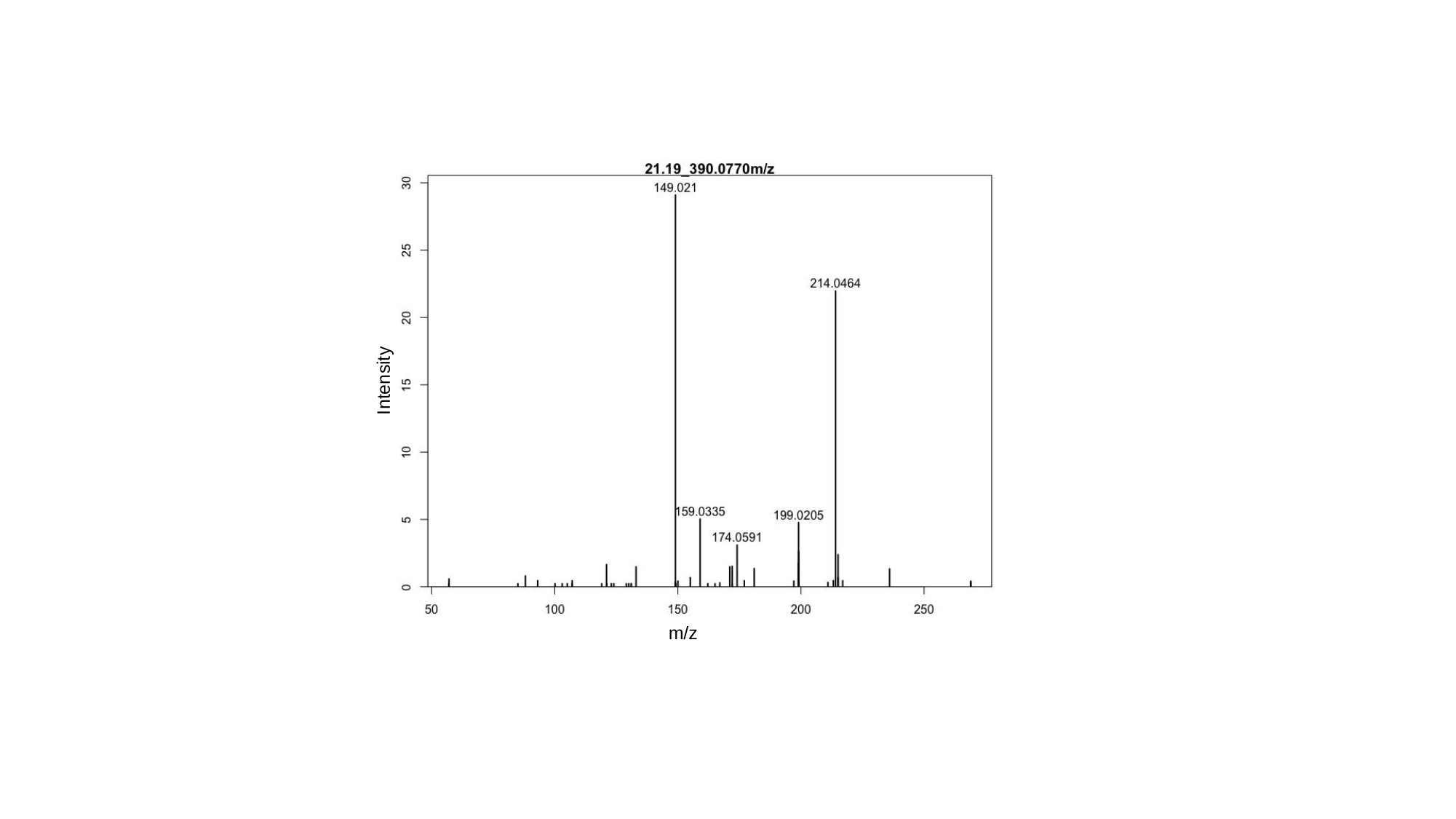

Intensity
m/z

## Slide 9
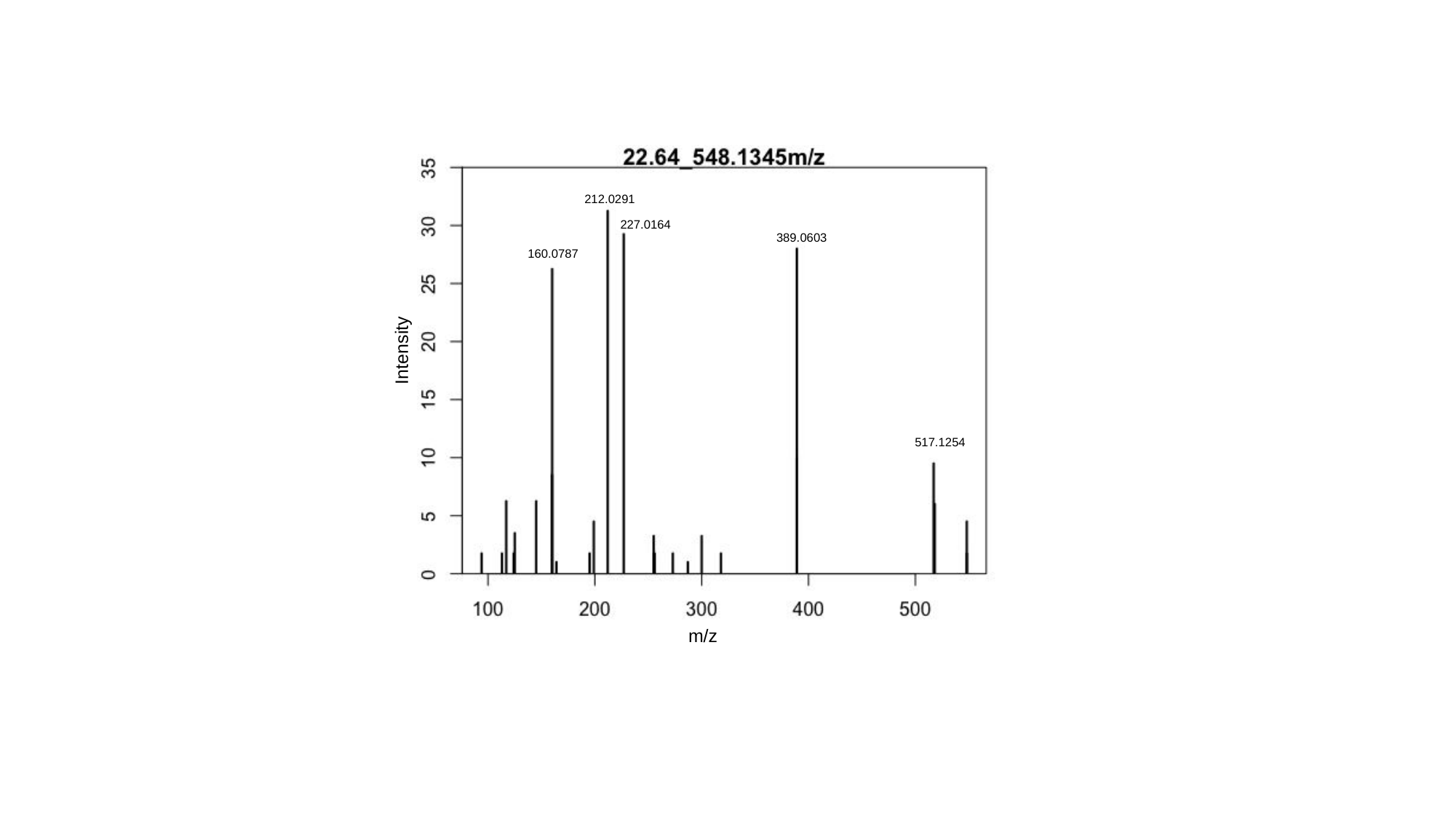

212.0291
227.0164
389.0603
160.0787
517.1254
Intensity
m/z

## Slide 10
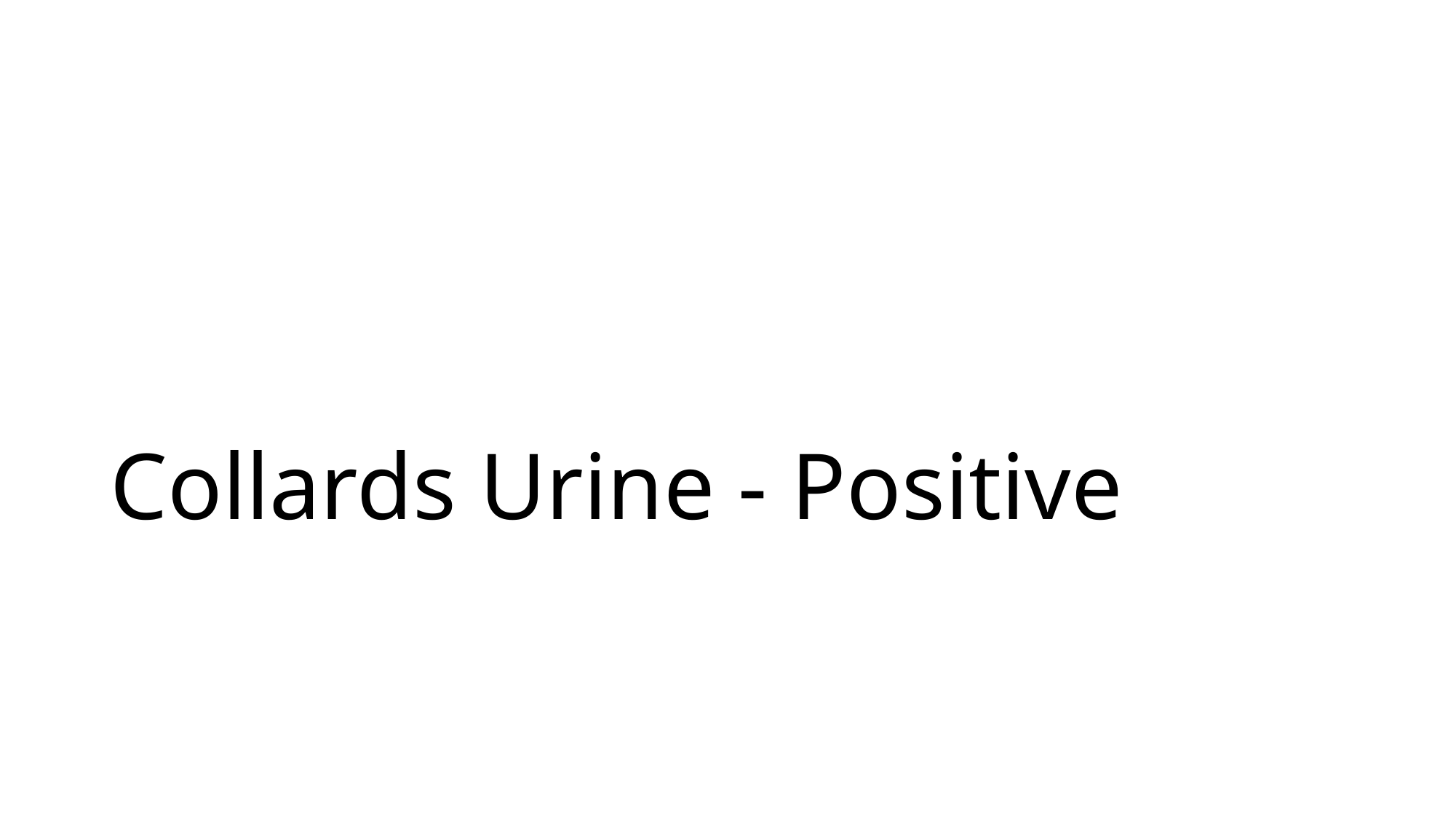

# Collards Urine - Positive

## Slide 11
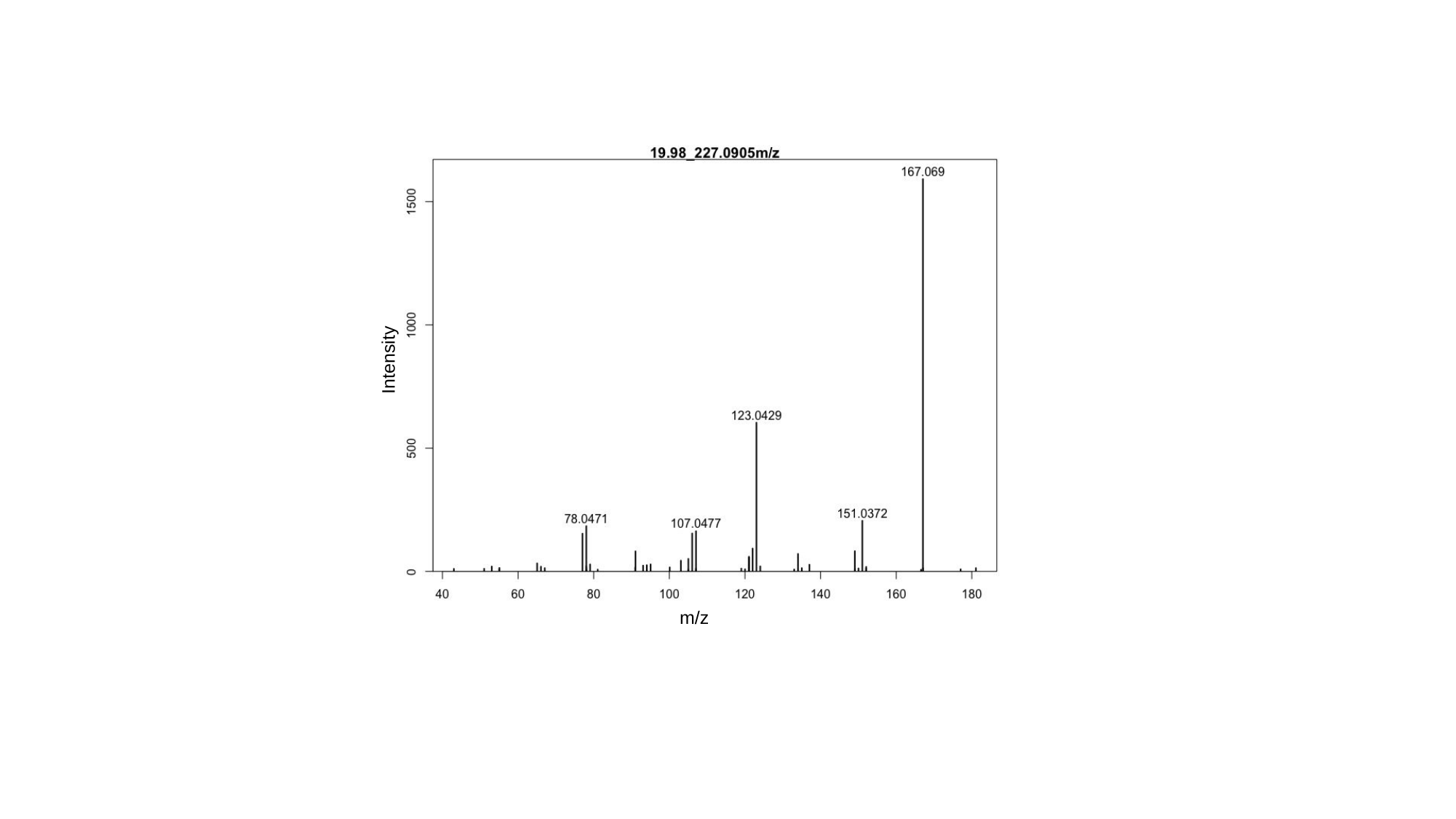

Intensity
m/z

## Slide 12
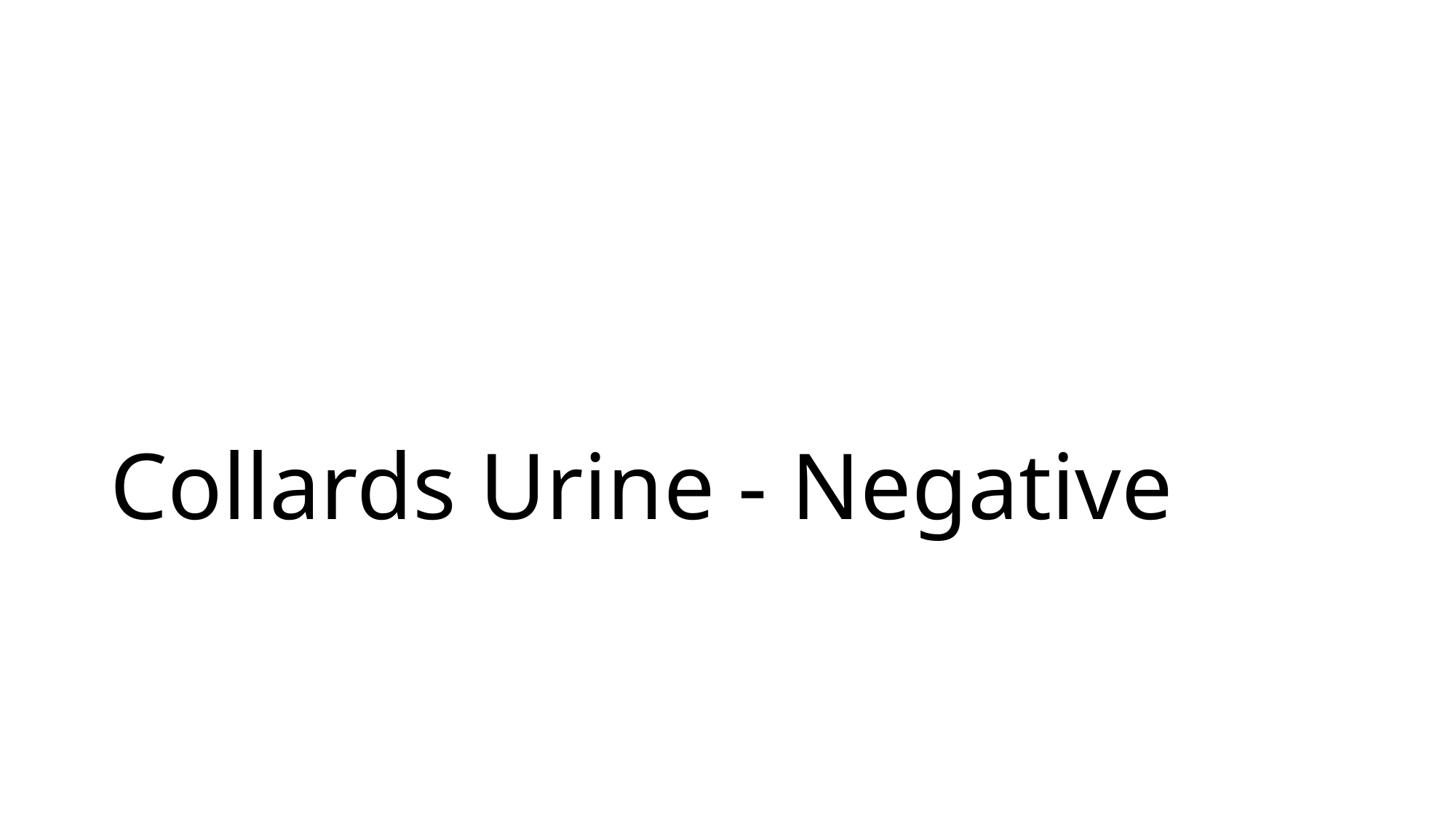

# Collards Urine - Negative

## Slide 13
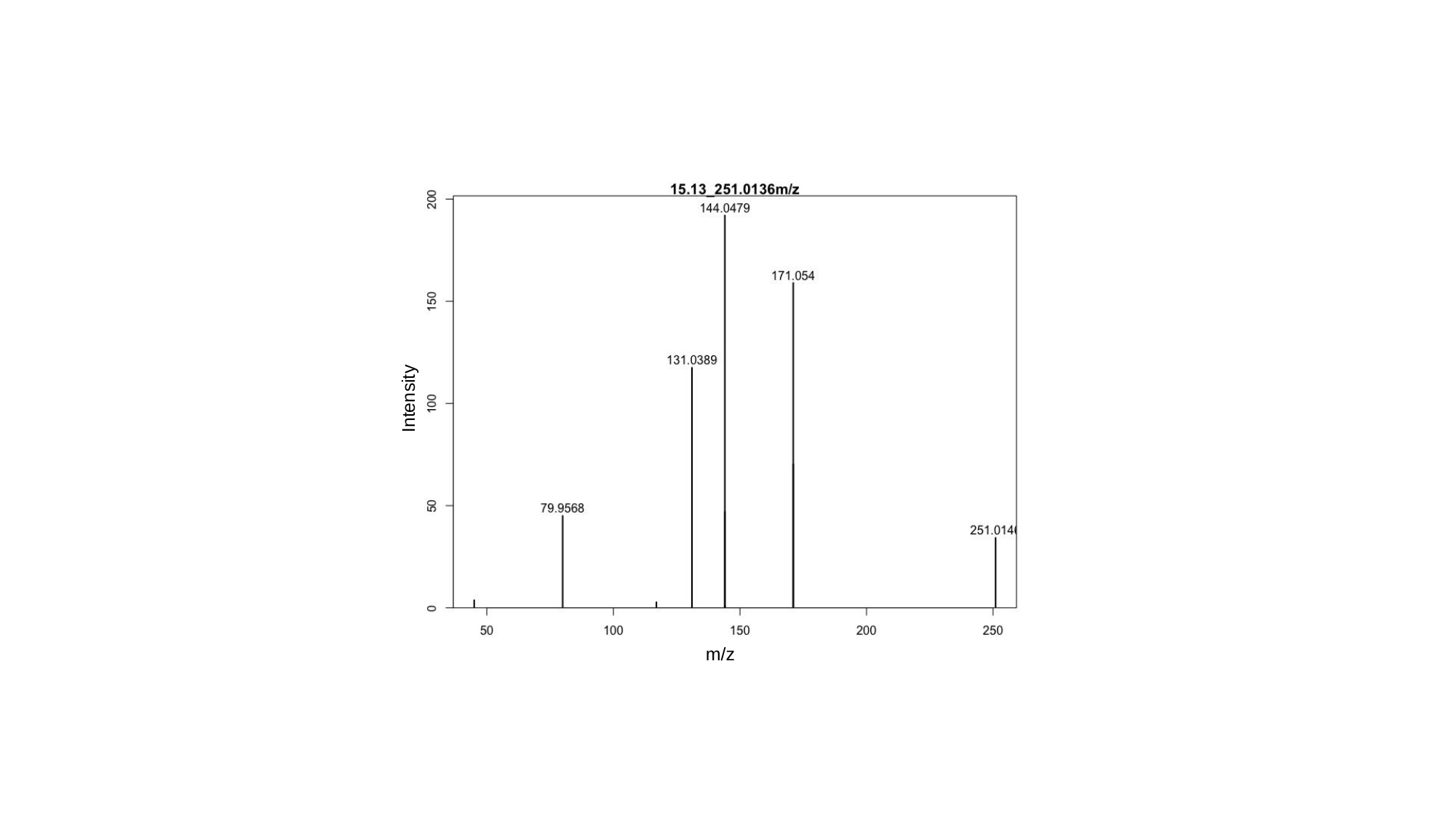

Intensity
m/z

## Slide 14
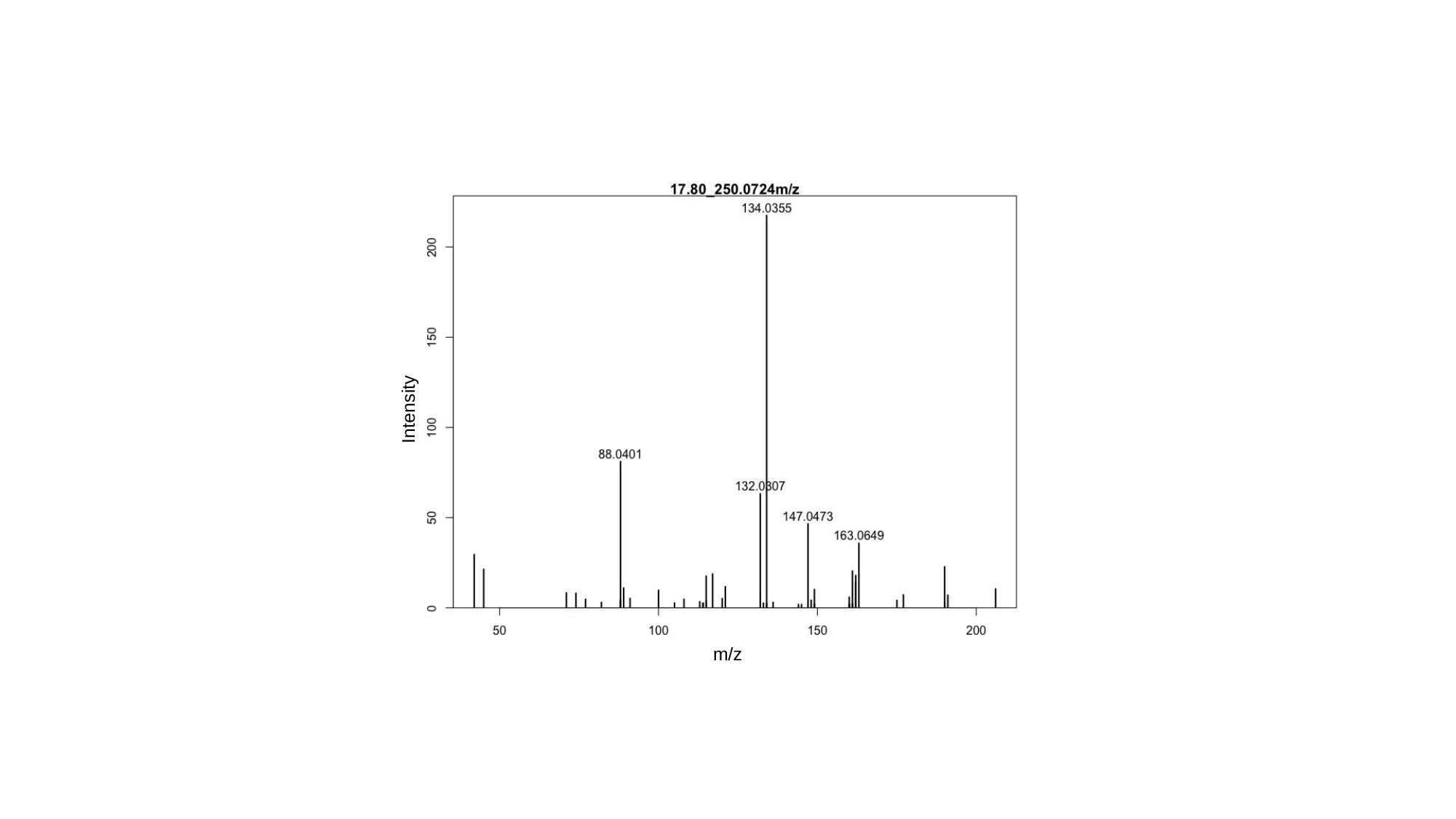

Intensity
m/z

## Slide 15
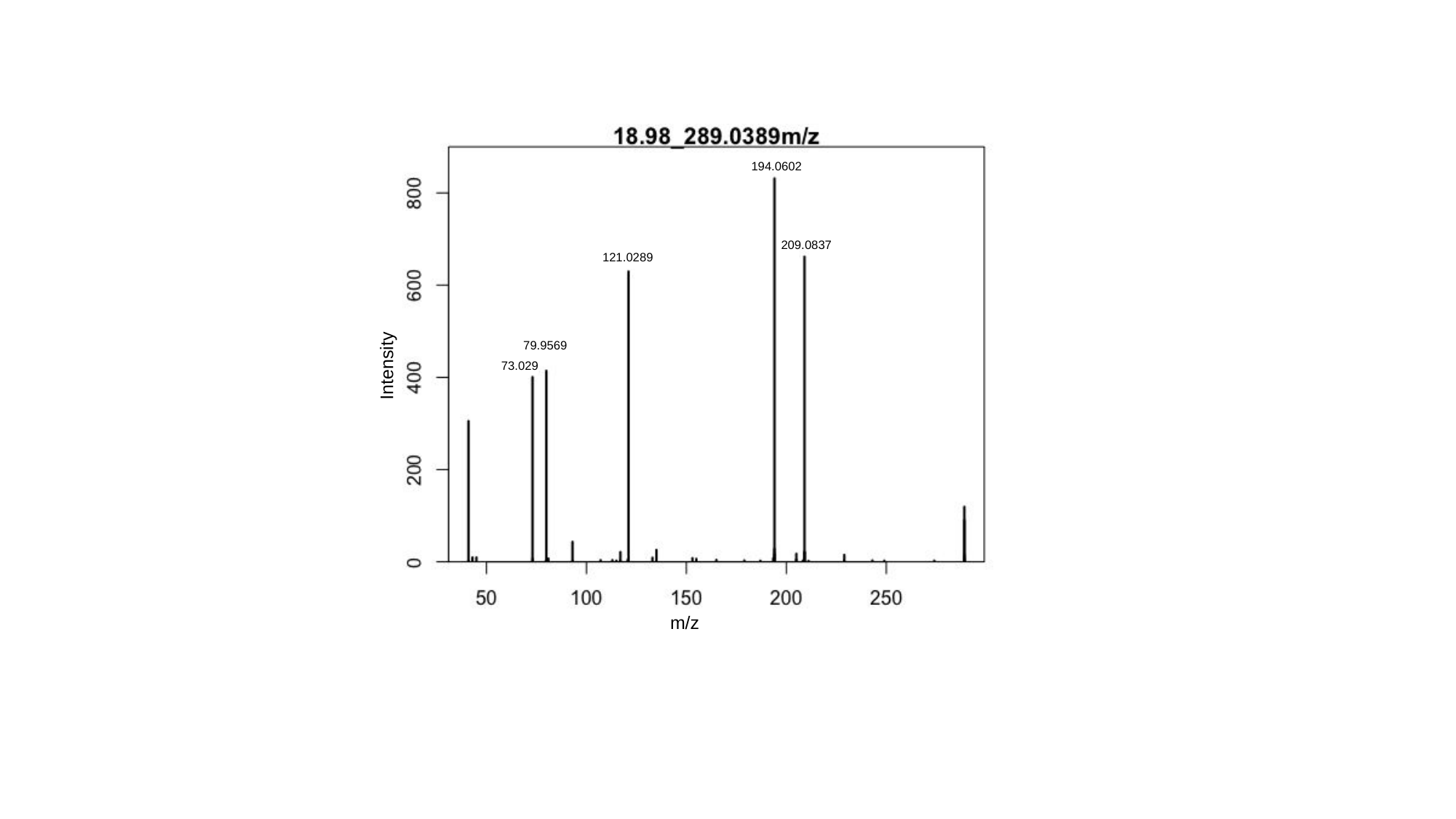

194.0602
209.0837
121.0289
79.9569
73.029
Intensity
m/z

## Slide 16
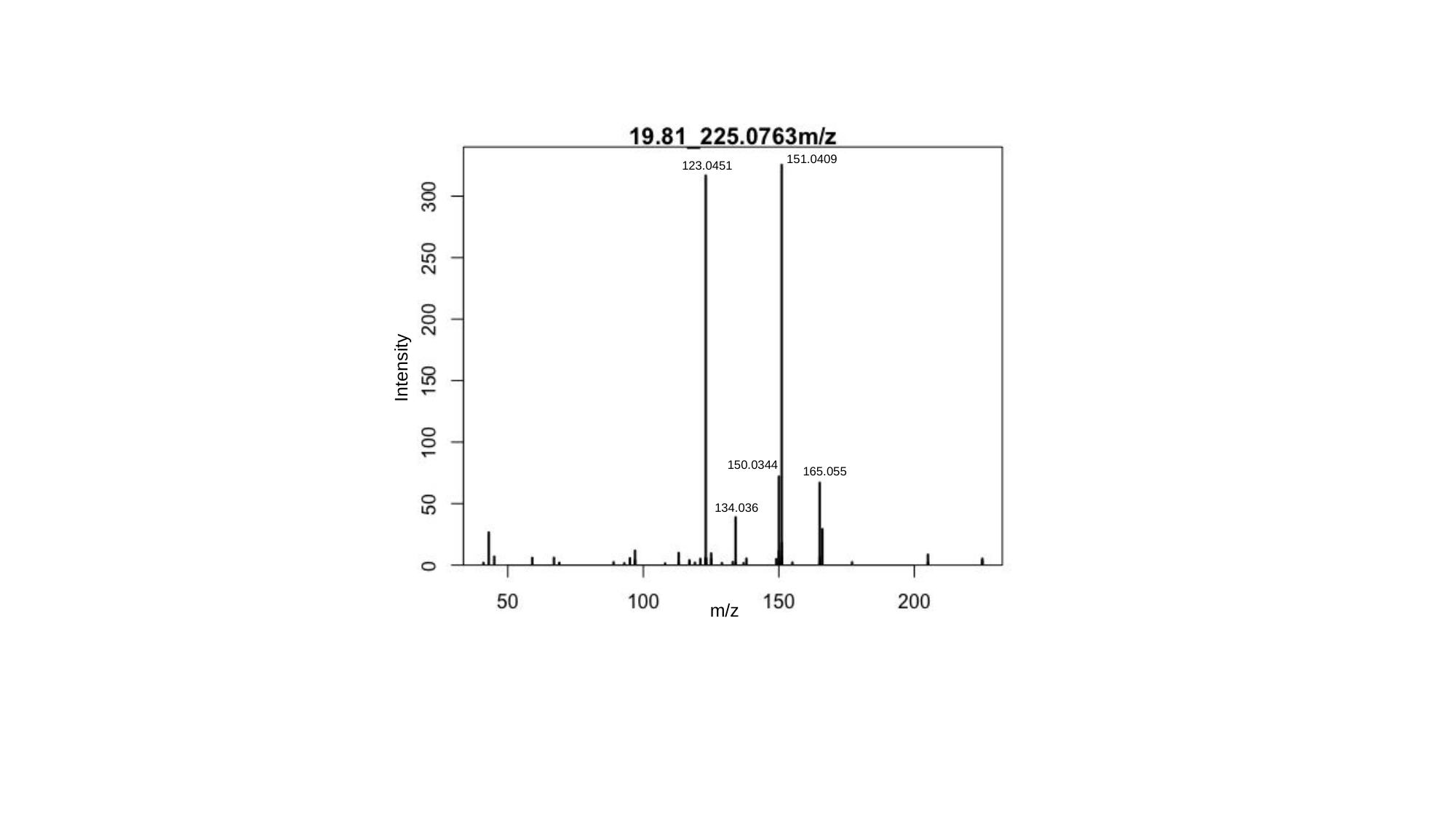

151.0409
123.0451
150.0344
165.055
134.036
Intensity
m/z

## Slide 17
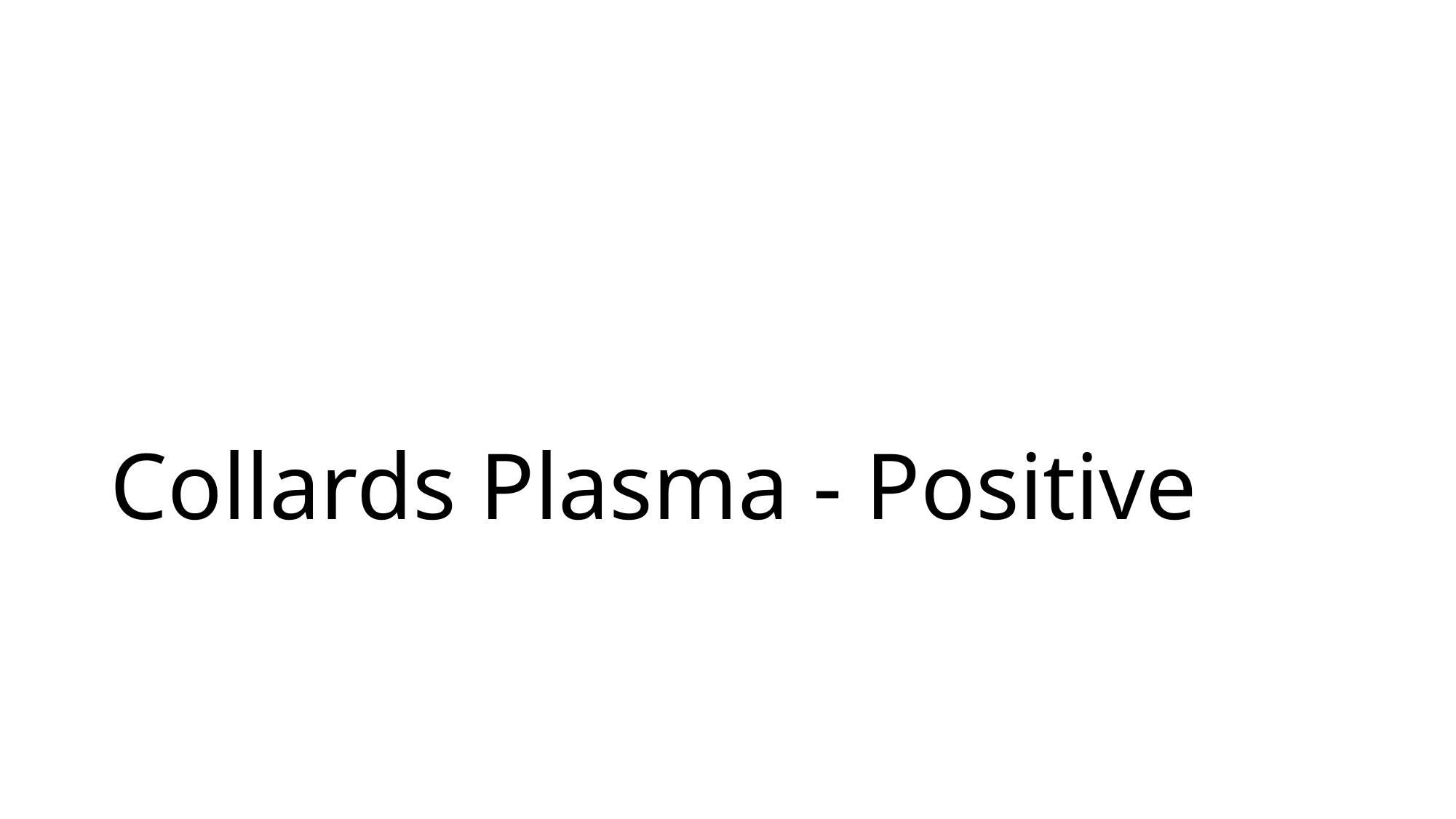

# Collards Plasma - Positive

## Slide 18
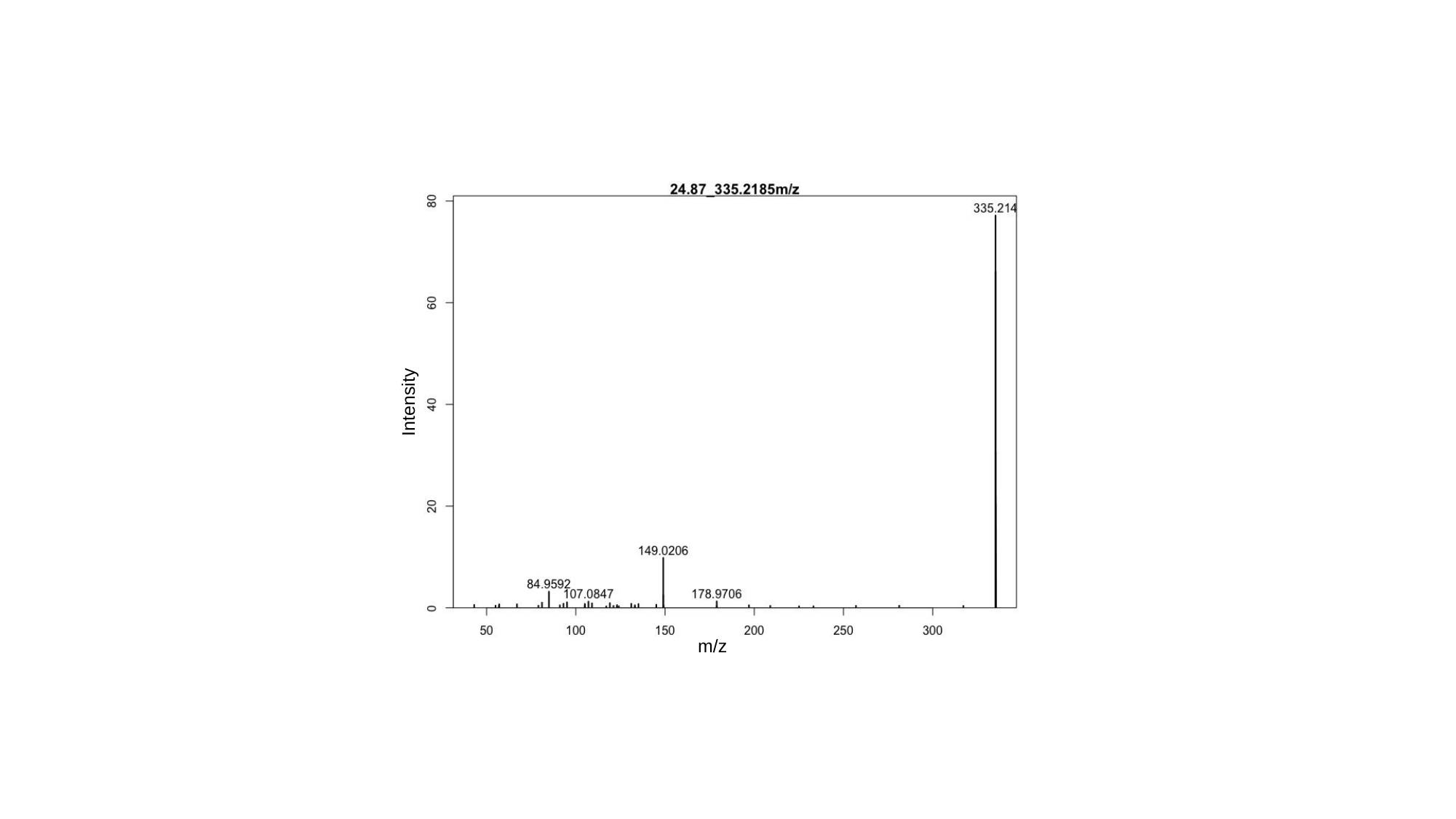

Intensity
m/z

## Slide 19
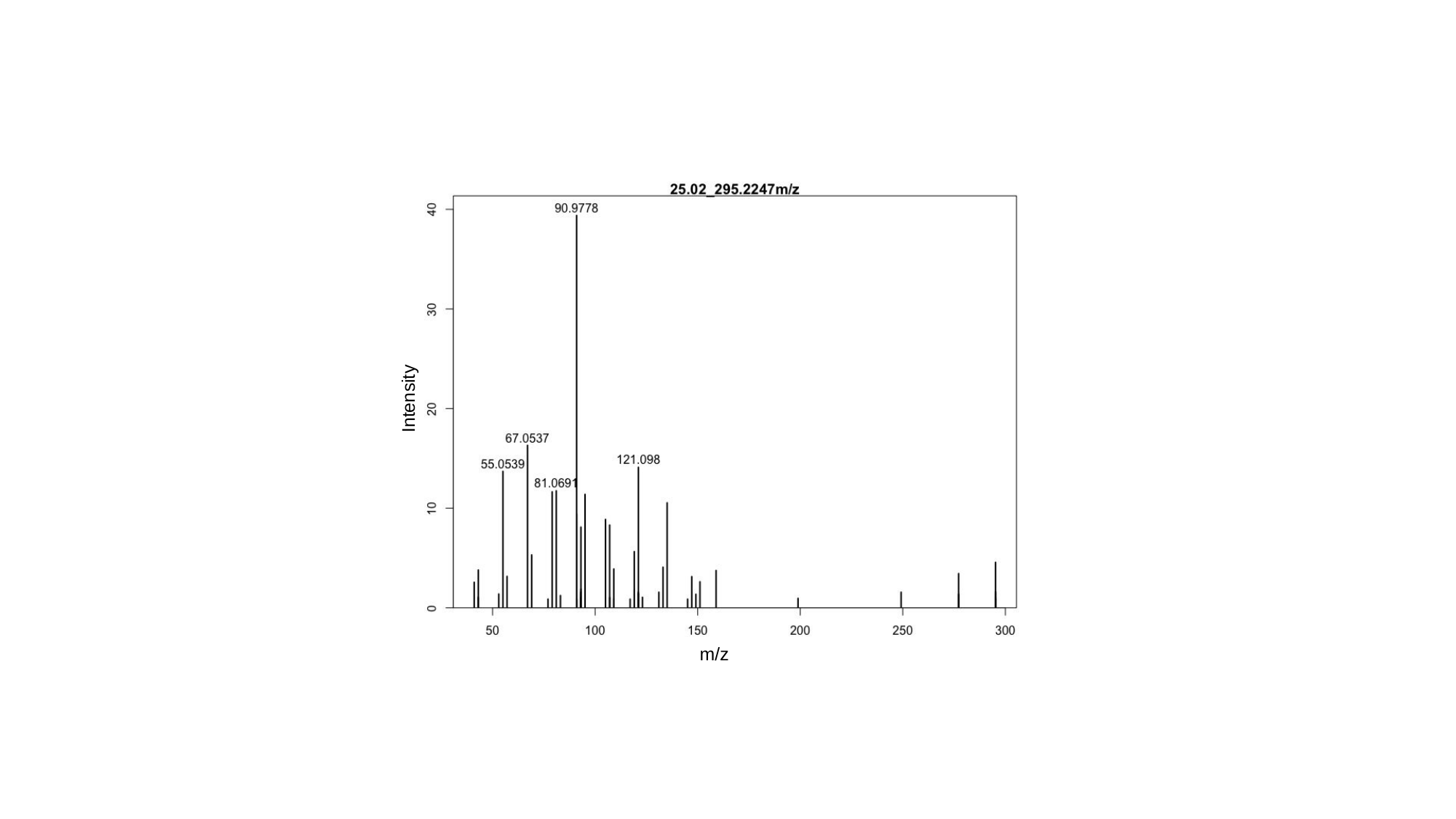

Intensity
m/z

## Slide 20
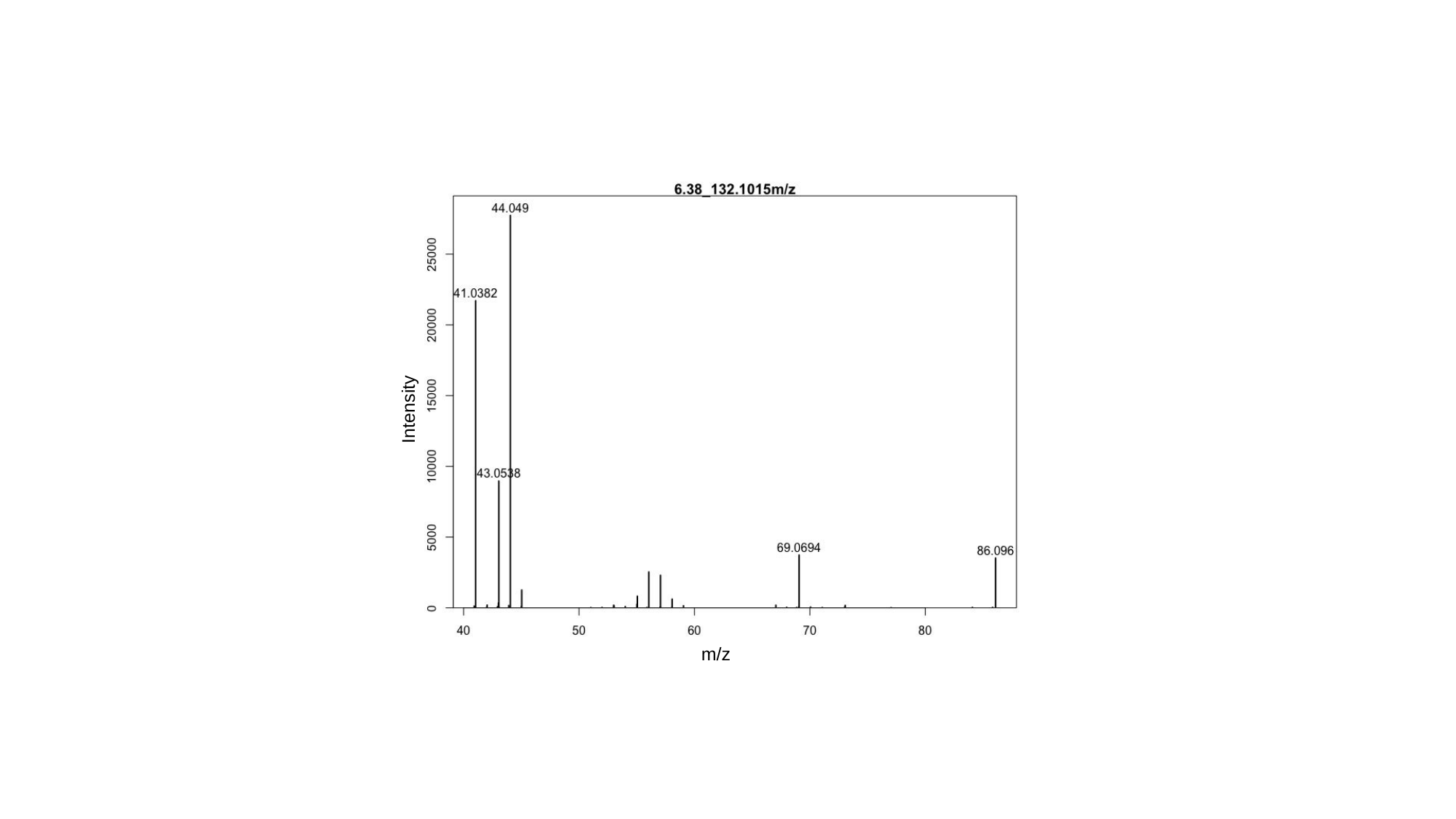

Intensity
m/z

## Slide 21
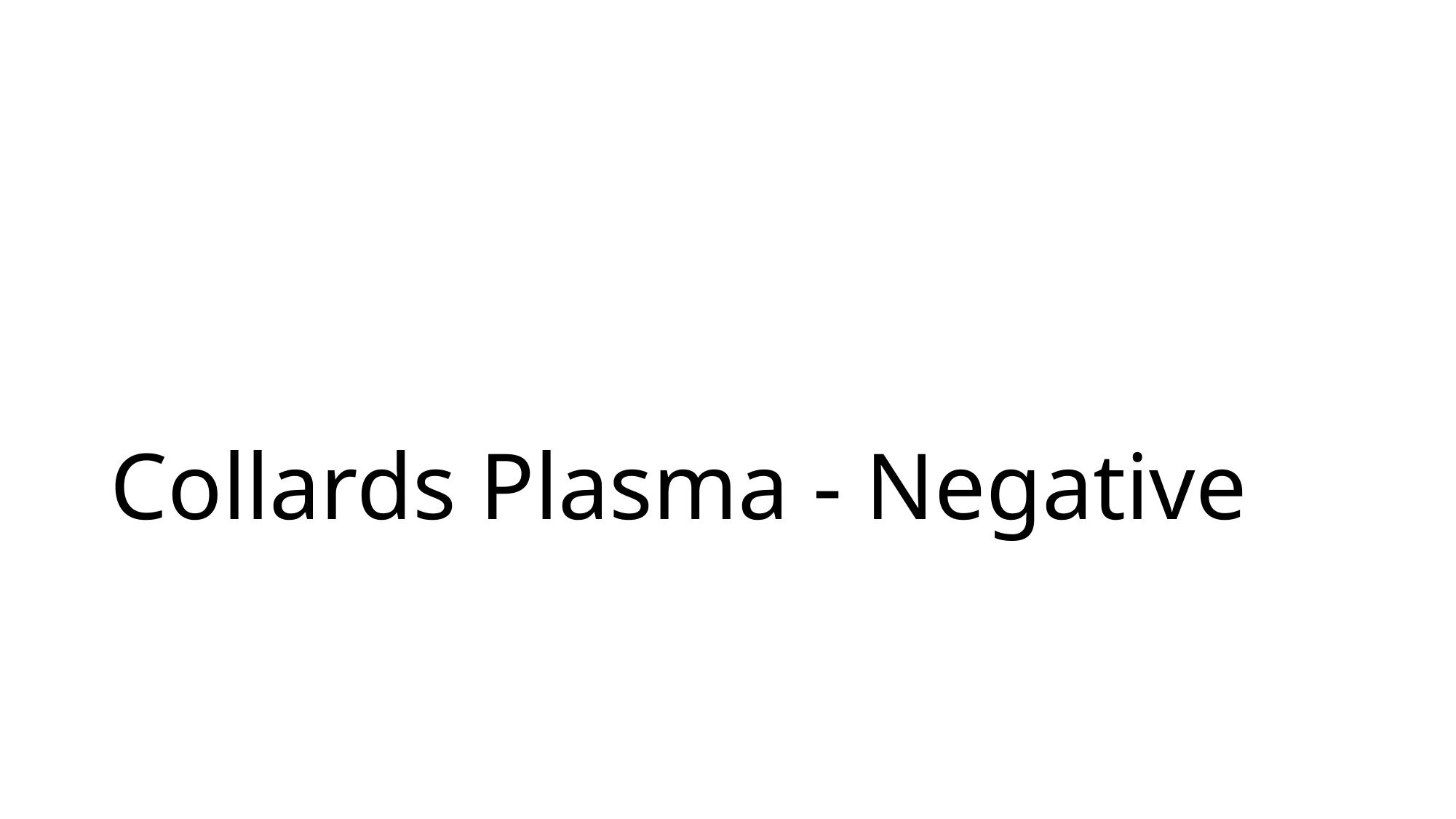

# Collards Plasma - Negative

## Slide 22
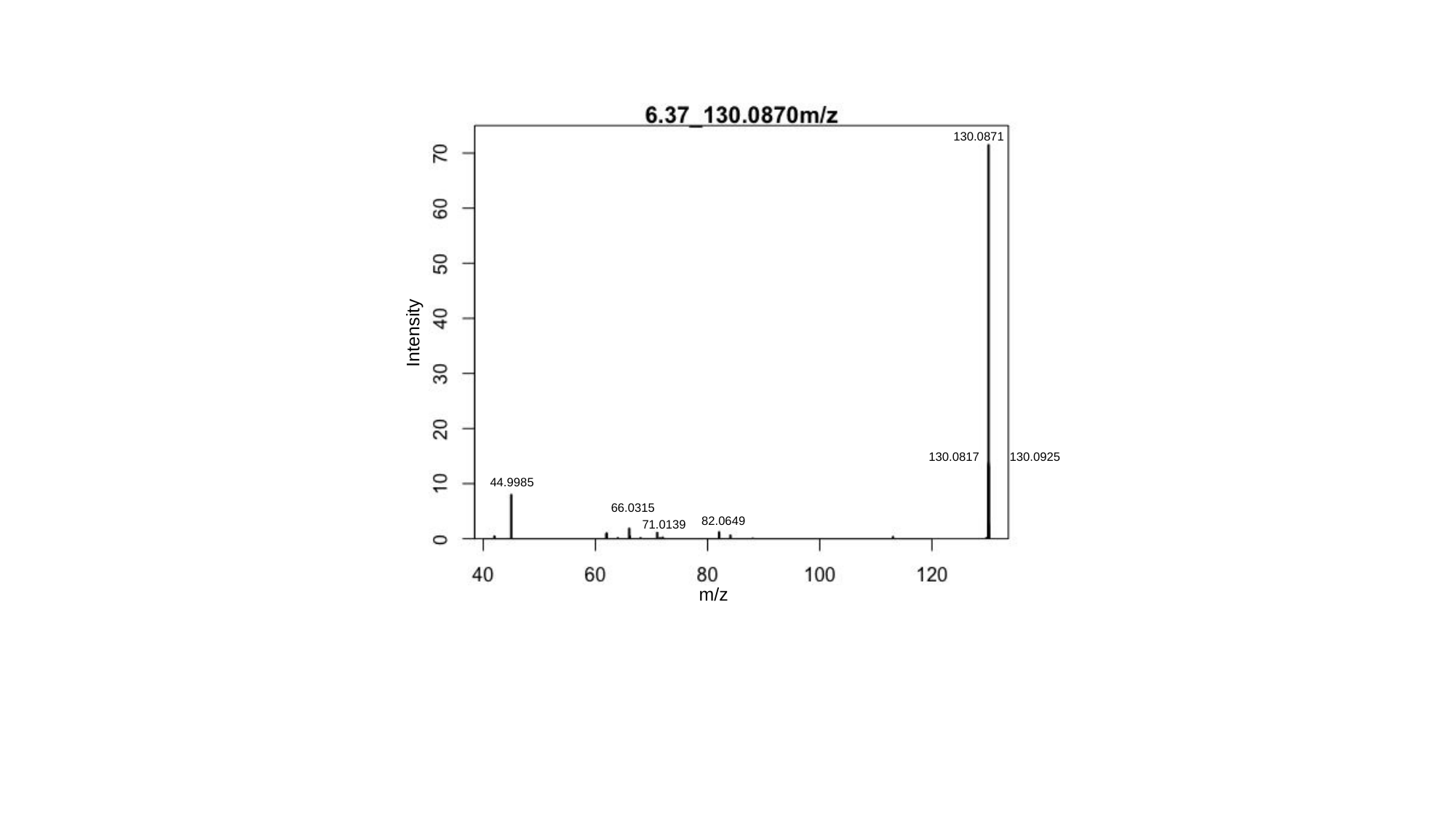

130.0871
130.0817
130.0925
44.9985
66.0315
82.0649
71.0139
Intensity
m/z
